# Machine learning prediction of bile salt export pump inhibition with Boruta feature selection in imbalanced data

**DOI:** 10.3389/fphar.2026.1911283

**Published:** 2026-09-09

**Authors:** Bailang Liu, Yong Liu, Hui Yang, Yang Li, Shanshan Yin, Junnan Wang, Zhikui Cheng, Xin Zhao, Xuejun Zhang, Shu Li, Lifei Liu

**Affiliations:** 1 Humanwell Healthcare (Group) Co, Ltd., Wuhan, Hubei, China; 2 Department of Infectious Diseases, Wuhan University, Wuhan, China

**Keywords:** BSEP inhibition, feature selection, imbalanced training, machine learning, QSAR modeling

## Abstract

**Introduction:**

Bile salt export pump (BSEP) inhibition is an important mechanistic risk factor for cholestatic drug-induced liver injury. Quantitative structure-activity relationship (QSAR) models may support early safety screening, but practical BSEP datasets are often limited in size and imbalanced.

**Methods:**

We evaluated a machine-learning workflow combining molecular descriptors and fingerprints, Boruta feature selection, and class-imbalance training. Multiple algorithms were assessed using cross-validation, a held-out test set, and an independent external validation set composed of compounds synthesized in an industrial research setting.

**Results:**

Tree-based models generally showed the strongest external performance. In particular, models using RDKit2D descriptors achieved Matthews correlation coefficients (MCC) above 0.65 and a ROC-AUC of 0.87, while several other tree-based model representation combinations achieved MCC values around 0.45-0.50. Boruta markedly reduced feature dimensionality, but its effect on predictive performance depended on both the molecular representation and learning algorithm; it was most beneficial for selected high-dimensional fingerprints and tree-based models, while offering limited or unfavorable effects in some support vector machine settings. Applicability-domain and SHAP analyses further revealed representation-dependent generalization patterns and highlighted contributions from lipophilicity, polarity, solubility, and molecular complexity.

**Discussion:**

Overall, the workflow maintained useful predictive performance under small and imbalanced conditions and provides a practical framework for early-stage BSEP risk screening.

## 1 Introduction

Drug-induced liver injury (DILI) remains one of the most critical safety concerns in drug development, which frequently leads to late-stage clinical failure and, in severe cases, post-marketing withdrawal of approved drugs (Babai et al., 2021). DILI can be categorized into hepatocellular, cholestatic, or mixed pattern (Ozturk et al., 2024). Recent studies have reported that cholestatic DILI accounts for approximately 10% to nearly 50% of all DILI cases, depending on patient population (Pinazo-Bandera et al., 2023). The basic pathological feature of cholestatic DILI is obstruction of bile flow and abnormal accumulation of bile acids in the liver. The accumulation of bile acids can induce hepatocellular toxicity through various mechanisms, including disruption of cell membrane structure, triggering organelle stress responses, and directly activating cell death pathways (Chiang and Ferrell, 2018; Jia et al., 2024). In this context, the bile salt export pump (BSEP), encoded by *ABCB11*, plays an important role in maintaining bile acid homeostasis. BSEP is an ATP-binding cassette transporter located on the canalicular membrane of hepatocytes and is responsible for the active efflux of bile salts from hepatocytes into bile (Ren et al., 2021). Any drug that interferes with BSEP function may form a significant pathogenic risk factor for cholestatic DILI. In fact, many known cholestatic hepatotoxic drugs have been shown to exert toxic effects through the inhibition of BSEP or other canalicular transporters (Jetter and Kullak-Ublick, 2020).

In the process of drug development, many experimental methods have been established to assess the inhibitory effects of candidate compounds on BSEP to eliminate candidate compounds with potential cholestasis risk. Commonly used detection methods include vesicle transport detection using membrane vesicles overexpressing human BSEPs; cell uptake or efflux detection based on hepatocyte-derived cell lines or transfection systems; and detection using human primary hepatocytes that more closely resemble the physiological bile acid processing environments (Kis et al., 2012). In recent years, human liver organoids and micro physiological systems have been explored as advanced *in vitro* platforms that more closely reproduce hepatic architecture and bile acid handling for cholestatic risk assessment (Zhang et al., 2023; Shao et al., 2025). Although these models are biologically relevant, they are limited by constraints such as low throughput, high cost, and sensitivity to experimental conditions. In this context, quantitative structure-activity relationship (QSAR) modeling based on machine learning (ML) or deep learning (DL) has received increasing attention as a complementary strategy for toxicity prediction, especially suitable for assessing the risk of DILI (Hong et al., 2017; 2018; Yan et al., 2022; Mostafa and Chen, 2023). By learning structure-toxicity relationships directly from existing datasets, ML-QSAR models enable rapid, cost-effective safety alerts prior to experimental testing.

Over the past two decades, many valuable explorations have been conducted in QSAR modeling of BSEP inhibition. Before ML methods were widely applied to QSAR modeling, Hirano et al. systematically analyzed key structural factors related to BSEP inhibition using linear regression models, providing early mechanistic clues for understanding transporter-related toxicity risks (Hirano et al., 2006). With the introduction of ML techniques into this field, early BSEP ML-QSAR studies primarily relied on molecular descriptors obtained from in-house or commercial software calculations to characterize the physicochemical properties and structural features of compounds (Warner et al., 2012; Montanari et al., 2016). In recent years, because of the accumulation of in-house BSEP inhibitor data, more robust machine learning models have been established. For example, McLoughlin et al. and Rodríguez-Pérez et al. trained QSAR models on datasets with relatively balanced class distributions and both achieved a Matthews correlation coefficient (MCC) of approximately 0.50 on the external validation set, demonstrating good generalization ability (McLoughlin et al., 2021; Rodríguez-Pérez and Gerebtzoff, 2021). Abdul Hameed et al. applied graph convolutional neural networks to BSEP inhibition modeling and obtained a ROC-AUC score of 0.86 on a test set with homology-based split (AbdulHameed et al., 2023), indicating that DL methods have potential in BSEP inhibitor classification. Despite the significant progress made in recent research, publicly available BSEP activity data still presents several challenges. First, although the number of BSEP potency experimental data is increasing, the overall dataset size remains limited compared to many other QSAR modeling datasets (Ryu et al., 2023; Sharma et al., 2023; Liu et al., 2025), easily introducing overfitting risks in high-dimensional molecular feature spaces. Second, the proportion of identified BSEP inhibitors in the public datasets is relatively low, leading to a significant imbalance in data distribution which increases the risk of false negatives and weakens the reliable detection of safety-critical positive compounds. These data-level limitations are actual problems encountered when using publicly available BSEP activity data for modeling. However, some previous BSEP modeling studies mitigated these limitations by incorporating proprietary in-house data. However, few studies have explicitly addressed the combined challenges of limited sample size and class imbalance when designing the modeling workflow. Moreover, the role of feature engineering in reducing redundant information and improving model stability has received relatively limited attention. These gaps provide an important opportunity for further systematic research on BSEP inhibition prediction.

In this study, we developed and evaluated a QSAR modeling workflow for BSEP inhibition prediction based on public BSEP activity data, with an explicit focus on practical modeling scenarios. Rather than optimizing a single best model, our workflow was designed to address key challenges, including limited sample size, high-dimensional feature spaces, and pronounced class imbalance. Specifically, we incorporated a tree-based feature-selection module to reduce feature redundancy and applied this strategy to two types of molecular representations: continuous molecular descriptors and discrete molecular fingerprints. We further examined how the effects of feature selection depended on the molecular representation and downstream learning algorithm, thereby evaluating whether dimensionality reduction improved or impaired external generalization under different modeling settings. Class-imbalance-aware learning was incorporated, and different modeling strategies were compared using cross-validation, a held-out test set, and an independent external validation set. Applicability-domain and feature-importance analyses were additionally used to relate predictive performance to chemical-space coverage and chemically meaningful molecular properties. Overall, this work aims to provide a robust and interpretable QSAR framework for BSEP inhibition prediction while offering practical guidance for method selection in other small-sample, imbalanced toxicity prediction tasks.

## 2 Methods

### 2.1 Study design

**FIGURE 1:**
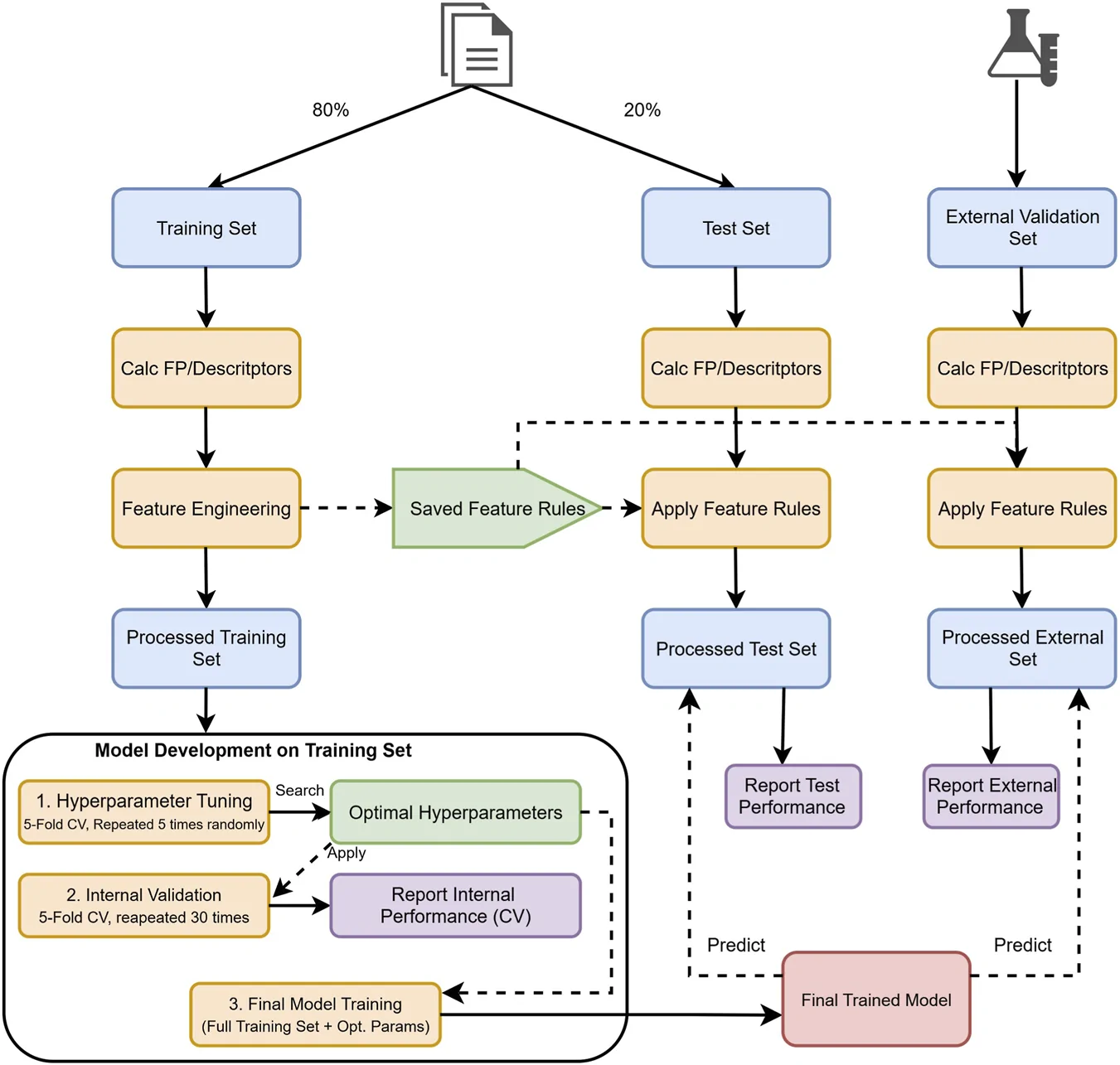
General workflow of the research.

The overall study design is illustrated in Figure 1. BSEP inhibitors and non-inhibitors were collected from published literature, and they were divided into a training set and a test set. Molecules tested in Humanwell Healthcare’s in-house assay were formed as an independent external validation test, to ensure that the constructed predicting model can be properly assessed on industrial data. During the data pre-processing stage, different molecular representations were generated for all molecules, followed by systematically feature filtering to reduce number of features and to enhance the stability of models. k-Nearest Neighbors (kNN), Support Vector Machine (SVM), Random Forest (RF) and Catboost were applied to construct QSAR models. Hyperparameter tuning was performed on the training set with cross-validation (CV). Performance regarding training set CV were reported to ensure the robustness of the model during the training stage. Next, optimized hyperparameters were chosen, and the full training set was used for training the final predicting models. Model performance on the test set and external validation set were evaluated for characterizing their generalization ability and stability across heterogeneous data sources. Finally, the interpretability and applicability domain of the models were evaluated.

### 2.2 Dataset preparation

BSEP inhibition data were acquired from three key literature sources: Warner et al. (2012), Morgan et al. (2013) and Sutherland et al. (2023), and their experimental measurements were consolidated into a unified dataset. Warner and Morgan evaluated concentration-dependent inhibition of human BSEP using membrane vesicles prepared from Sf21 and Sf9 insect cells, respectively. In both studies, ATP-dependent [^3^H]taurocholate transport was measured, and IC_50_ values were derived from concentration-response data. Sutherland used a radioactivity-based human *ABCB11* incorporation assay with filtration and liquid-scintillation counting and reported AC_50_ values. Although these assays measured the same functional endpoint of concentration-dependent inhibition of human BSEP-mediated transport, differences in vesicle preparations, assay conditions, and analytical procedures may affect absolute potency estimates. After combining the datasets, we followed the labelling strategy recommended by International Transporter Consortium workflow on BSEP inhibition in drug discovery (Kenna et al., 2018). Compounds with IC_50_ (or AC_50_) ≤ 25 μM were labeled as inhibitors and IC_50_ ≥ 100 μM were labeled as non-inhibitors, with intermediate cases excluded. Because the assays were not analytically identical, the potency values were used only for this conservative categorical assignment and were not treated as strictly interchangeable continuous measurements. The external validation dataset consisted of compounds synthesized during internal drug discovery programs at Humanwell Healthcare. Their BSEP inhibitory activities were experimentally determined using membrane vesicles expressing recombinant human BSEP. Compounds were tested at eight concentrations ranging from 0.512 to 200 μM, using sodium taurocholate as the probe substrate under parallel ATP and AMP-containing conditions. Vesicle-associated taurocholate was quantified by LC-MS/MS, and concentration-response data were used to derive IC_50_ values. Further experimental details are provided in the Supplementary Material. These compounds and their experimental measurements were not used during model training, feature selection, or hyperparameter optimization. The internally generated IC_50_ values were converted into binary activity labels using the same thresholds applied to the literature-derived dataset above.

The Simplified Molecular Input Line Entry System (SMILES) describes the structure of molecules using short ASCII string. SMILES representations for all curated molecules were obtained through PubChem public databases. Our entire dataset was standardized using RDKit 2025.3.3 (Landrum et al., 2025), to ensure consistent structural representations across different data sources, and improving the stability of following QSAR modeling. The standardization pipeline involved parsing and cleaning the original structures, unifying the functional group representations and charge states, selecting the parental chemical entity in multi-fragment molecules, and removing counterions or solvent residues. Additional steps included removing molecules containing elements other than ten common elements (H, C, N, O, P, S, F, Cl, Br, I), neutralization and tautomer canonicalization to yield stable and chemically coherent representations. Finally, in this study, stereochemical annotations were removed, and unique canonical SMILES for each compound were generated. This systematical standardization workflow provides a robust foundation for subsequent feature generation and model development.

Compounds that appeared multiple times across different literature sources were consolidated using a consensus-based deduplication procedure. When the labels of the same compounds exhibited contradictory classifications across different literature sources, it was considered as a conflict case and excluded from modeling dataset to prevent inconsistencies. For compounds having the same classification label across different sources, only one record was retained. In total, 90 compounds were excluded because of inter-source label conflicts. Finally, a stratified sampling procedure was applied to divide the dataset into a training set and a test set with the split ratio 4:1 while preserving the proportion of positive and negative compounds. Humanwell Healthcare’s in-house tested compounds were kept as an independent external validation set. The numbers of positive and negative compounds in all three datasets: the training set, test set, and external validation set are summarized in Table 1.

**TABLE 1:** Number of BSEP inhibitors and non-inhibitors in the training set, test set, and external validation set.

BSEP classification	Training set	Test set	External validation set
Inhibitors	166	41	48
Non-inhibitors	661	166	32
Total	827	207	80

### 2.3 Molecular representation and feature selection

In this study, four molecular representation methods were employed to encode the chemical structures of all compounds: Avalon fingerprints (Gedeck et al., 2006), PubChem fingerprints (Kim et al., 2016), Mordred descriptors (Moriwaki et al., 2018), and RDKit2D descriptors (Yang et al., 2019). The first two belong to discrete molecular fingerprints, which capture the presence or absence of specific structural fragments or subpatterns by mapping them into fixed-length binary vectors. The latter two are continuous molecular descriptors that quantitatively characterize topological, physicochemical, and geometric properties of molecules using real-valued feature vectors. Scikit-fingerprints software package 1.17 (Adamczyk and Ludynia, 2024) was used for generating molecular representations.

For the discrete molecular fingerprints, a feature filtration pipeline was constructed to obtain a stable and informative feature subset. First, bits with a frequency of one below 1% or above 99% were removed to eliminate extremely sparse features that contain minimal discriminatory information. Univariate selection was then performed using mutual information, retaining the top 500 bits most strongly associated with the class label. Mutual information $I\left(X;Y\right)$ quantifies the shared information between a feature *X* and label *Y*, which is defined in Equation 1:

$$I\left(X;Y\right)=\sum_{x}\sum_{y}p\left(x,y\right)\log\frac{p\left(x,y\right)}{p\left(x\right)p\left(y\right)}$$ (1)

where $p\left(x,y\right)$ referred as the joint probability distribution of a feature *X* taking value *x* and a class label *Y* taking value *y*. $p\left(x\right)$ and $p\left(y\right)$ represent marginal probability distribution of the feature *X* and label *Y*, respectively. The Higher $I\left(X;Y\right)$ indicates feature *X* could provide more efficient information to distinguish positive and negative labels. To further refine the feature set, we applied the Boruta algorithm (Kursa et al., 2010): a model-based “all-relevant” feature selection method aiming at identifying all features that contribute to prediction meaningfully. To achieve this, Boruta creates “shadow features” for each of original features, whose values are randomly permuted. A random forest model is then trained as base model on the combined set of real and shadow features, and feature-importance scores are computed for all. For each original feature, its importance is repeatedly compared to the shadow feature, and the original feature will only be accepted if it outperforms the shadow feature across multiple iterations. To further enhance the stability of Boruta feature selection, a sampled Boruta procedure was adopted: 80% of molecules in the training set were selected *via* stratified sampling in each iteration, Boruta was repeated 30 times, and features accepted in at least 21 runs (≥70%) were retained as the final set. All feature selection steps were conducted exclusively on the training set. The test and external validation sets inherited the selected feature columns from the training set.

For the continuous molecular descriptors, the corresponding feature filtration pipeline was introduced here. Descriptor columns containing missing values and features with constant values across all molecules were first removed. Then, Pairwise Pearson correlation coefficients were computed, which is defined in Equation 2:

$$r_{ij}=\frac{\sum_{k=1}^{n}\left(x_{k,i}-{\bar{x}}_{i}\right)\left(x_{k,j}-{\bar{x}}_{j}\right)}{\sqrt{\sum_{k=1}^{n}{\left(x_{k,i}-{\bar{x}}_{i}\right)}^{2}}\sqrt{\sum_{k=1}^{n}{\left(x_{k,j}-{\bar{x}}_{j}\right)}^{2}}}$$ (2)

where $x_{k,i}$ denotes the value of descriptor *i* for molecule *k*, and ${\bar{x}}_{i}$ is its mean value. When $\left|r_{ij}\right|>0.90$ , one of the descriptors was removed due to excessive redundancy. To normalize descriptors measured on different scales, we applied standard scaler, which transforms each feature according to Equation 3:

$$x^{\prime }=\frac{x-\mu }{\sigma }$$ (3)

where $x^{\prime }$ is the scaled value, *x* is the raw value, μ is the mean value of the feature and σ is the standard deviation. The sampled Boruta procedure, which was introduced for discrete fingerprints, was applied here subsequently. The test set and external validation set kept the same feature columns selected from the training set and were standardized using the training set mean and standard deviation values.

### 2.4 Model development

BSEP inhibitor prediction models were built using four machine learning algorithms with Scikit-learn 1.6.1 software package (Pedregosa et al., 2011): kNN (Cover and Hart, 1967), SVM (Cortes and Vapnik, 1995), RF (Breiman, 2001) and CatBoost 1.2.8 software package (Prokhorenkova et al., 2019). kNN algorithm classifies a compound based on the labels of its closest neighbors in feature space, reflecting local structural similarity. SVM constructs a hyperplane that maximizes the margin between classes and can incorporate kernel functions to model nonlinear structure-activity relationships. RF is an ensemble of decision trees trained on randomly sampled features and data subsets, providing robust predictions through majority voting and reduced variance. CatBoost is a gradient boosting decision tree method that employs ordered boosting and symmetric tree structures to mitigate prediction bias and effectively capture complex feature interactions. These models, whose architecture varied from simple to complex, provide a more comprehensive comparison of how different learning patterns perform in predicting BSEP inhibition.

To handle the class imbalance between BSEP inhibitors and non-inhibitors, class reweighting approach was applied to SVM, RF and CatBoost classifiers. A weight *w*_*c*_ is assigned to each class *c* as shown in Equation 4:

$$w_{c}=\frac{N}{2N_{c}}$$ (4)

where N is the total number of molecules in the training set, and N_c_ is the number of molecules in class c. In SVM, this weight affects the loss function directly and further guides the decision boundary. In RF and Catboost, this weight influences the tree-splitting criteria or boosting loss, and increases the importance assigned to minority class.

During the hyperparameter optimization of each algorithm, we adopted a repeated five-fold CV strategy. Specifically, the training set was randomly divided into five mutually exclusive subsets in each repetition, with four used for model construction and the remaining one for validation. By alternating between different subsets as the validation set, a complete five-fold evaluation result can be obtained. To reduce the randomness of a single random partition, this five-fold process was repeated five times and the Matthews correlation coefficient (MCC) obtained from all repetitions was averaged to estimate the overall performance of the hyperparameter set. Finally, the parameter combination with the highest average MCC was considered the optimal setting for the algorithm and used to train the final model on the complete training set.

The hyperparameters were tuned with grid search method. For kNN, the parameters n_neighbors (k = 3, 5, 7 and 9) and weights (“uniform”, “distance”) were optimized. For SVM, a radial basis function kernel was employed and the regularization parameter C (0.01, 0.1, 1, 10 and 100) and kernel width gamma (0.0001,0.001,0.01,0.1 and 1.0) were optimized. For RF, n_estimators (200, 400 and 800 trees), min_samples_leaf (2, 5 and 10), min_samples_split (2, 5) and max_depth (None, 10) were optimized. For CatBoost, learning_rate (0.03, 0.05), iterations (400, 800), depth (4, 6 and 8) and L2 regularization parameter (1.0, 5.0 and 10.0) were optimized. For all models, except for parameters mentioned here, default values were set for other parameters.

### 2.5 Model evaluation and performance metrics

Model performance was evaluated on the training set, test set and external validation set. For the training set, a 30x5-fold CV procedure was applied to assess model robustness. In each repetition, the training data were stratified separated into five exclusive folds, and four of them were used to train the model, while the remaining one-fold was used for evaluating the prediction. This five-fold cycle was repeated 30 times using different random seeds, to provide a stable estimate of model behavior. Final model was trained with the complete training set with the optimal hyperparameters, and it was used to predict the test and external validation set. Performance on the test set reflects the model’s generalization performance on data drawn from the same distribution but not seen during training, while the external validation set further examines the extrapolation ability of the model to an independent dataset, evaluating its reliability for real-world applications.

Accuracy, sensitivity (true positive rate, TPR), specificity (true negative rate, TNR), balanced accuracy, MCC and area under the receiver operating characteristic curve (ROC-AUC) were used to measure model performance. These metrics were derived by comparing model predictions with actual BSEP inhibition data. They were calculated using Equations 5-11:

$$\text{Accuracy}=\frac{\text{TP}+\text{TN}}{\text{TP}+\text{TN}+\text{FP}+\text{FN}}$$ (5)

$$\text{Sensitivity}=\frac{\text{TP}}{\text{TP}+\text{FN}}$$ (6)

$$\text{Specificity}=\frac{\text{TN}}{\text{TN}+\text{FP}}$$ (7)

$$\text{Balanced Accuracy}=\frac{\text{Sensitivity}+\text{Specificity}}{2}$$ (8)

$$\text{MCC}=\frac{\text{TP}\times \text{TN}-\text{FP}\times \text{FN}}{\sqrt{\left(\text{TP}+\text{FP}\right)\left(\text{TP}+\text{FN}\right)\left(\text{TN}+\text{FP}\right)\left(\text{TN}+\text{FN}\right)}}$$ (9)

$$\text{ROC}-\text{AUC}=\int_{0}^{1}\text{TPR}\left(\text{FPR}\right)d\left(\text{FPR}\right)$$ (10)

FPR=1−TNR (11)

where TP, TN, FP and FN represent true positives, true negatives, false positives and false negatives, respectively. FPR refers to false positive rate.

To quantify the sampling uncertainty of the test and external validation performance estimates, stratified nonparametric bootstrap resampling was performed. Positive and negative compounds were separately resampled with replacement while preserving the original class counts, and the six performance metrics were recalculated for each resample. This procedure was repeated 5,000 times for each model, and the 2.5th and 97.5th percentiles of the bootstrap distributions were used as the lower and upper limits of the 95% confidence intervals.

### 2.6 Applicability domain

For models developed using continuous molecular descriptors, the applicability domain (AD) was defined using a range-based boundary approach derived from the training set. The range comparison was performed using standardized descriptor values; the test and external validation sets were transformed using the corresponding training-set means and standard deviations before comparison.

Specifically, for the *i*th descriptor, the individual distance contribution *d*_*i*_ was defined as shown in Equation 12:

$$d_{i}=\left\{\begin{array}{l} 0,\\ x_{i}^{\min}-x_{i},\\ x_{i}-x_{i}^{\max}, \end{array}\right.\;\begin{array}{c} x_{i}^{\min}\leq x_{i}\leq x_{i}^{\max}\\ x_{i}<x_{i}^{\min}\\ x_{i}>x_{i}^{\max} \end{array}$$ (12)

where *x*_*i*_ denotes the value of the i th descriptor for the query compound, $x_{i}^{\min}$ and $x_{i}^{\max}$ represent the boundary values of that descriptor in the training set. The overall distance of a compound with *n* descriptors to the AD was then defined as shown in Equation 13:

$$D\left(x\right)=\sqrt{\sum_{i=1}^{n}d_{i}^{2}}$$ (13)

A compound with *D*(*x*) = 0 was considered to be fully inside the AD. Non-zero distance indicates that the compound exceeds the training boundaries in at least one descriptor dimension.

For models developed using discrete molecular fingerprints, the AD was defined using the nearest-neighbor maximum Tanimoto similarity. The binary Tanimoto similarity between a query compound *x* and a training compound *t* was calculated as shown in Equation 14:

$$T\left(x,t\right)=\frac{c}{a+b-c}$$ (14)

where a and b denote the numbers of active bits in *x* and *t*, respectively and *c* is the number of shared active bits. For each compound *x*, the AD score was defined as the maximum similarity to the training set, as shown in Equation 15:

$$s\left(x\right)=\max_{t\in \text{Train}}T\left(x,t\right)$$ (15)

The threshold τ was determined as the 5^th^ percentile of the NN-max similarity distribution calculated within the training set. Compounds with $s\left(x\right)\geq \tau$ were considered as falling within the AD.

To provide a physicochemical overview of the chemical space covered by each dataset, molecular weight (MW), calculated logP (cLogP), topological polar surface area (TPSA), numbers of hydrogen-bond donors (HBD) and acceptors (HBA), and rotatable bonds (RotB) were calculated using RDKit. To complement the fingerprint-based nearest-neighbor similarity analysis, scaffold overlap was evaluated using Bemis-Murcko scaffolds generated from the standardized molecular structures. The number of unique scaffolds was counted separately for the test and external validation datasets. Each compound in these datasets was then classified as training-shared when its scaffold was present in the training set or as unseen when its scaffold was absent from the training set.

### 2.7 Feature importances

To enhance interpretability while minimizing dependence on model-specific assumptions, SHAP (SHapley Additive exPlanations) was used as a model-agnostic framework to quantify feature importance in current modeling tasks. Globally, aggregating SHAP value distributions across compounds yields a robust feature-importance ranking and reveals whether a feature’s effect is consistently directional or varies across chemical subspaces. Locally, SHAP decomposes a single compound’s prediction into feature-wise contributions, clarifying why the model classifies it as a likely inhibitor or non-inhibitor. The additive explanation form was adopted as shown in Equation 16:

$$f\left(x\right)=E\left[f\left(X\right)\right]+\sum_{i=1}^{n}\phi_{i}$$ (16)

where $f\left(x\right)$ is the model output for compound *x*, $E\left[f\left(X\right)\right]$ is the baseline output derived from the tree-based model structure and the training data distribution *via* weighted expectations over decision paths. *n* is the number of features and $\phi_{i}$ is the SHAP contribution of feature *i* for *x*. This decomposition enables a link between predictions and features. The SHAP was calculated by the SHAP software package 0.50.0 (Lundberg and Lee, 2017).

## 3 Results

### 3.1 Model performance

As shown in Table 1, the BSEP inhibition prediction task in this study was characterized by a limited sample size and an imbalanced class distribution. To evaluate model performance under these data conditions, we applied the proposed workflow, including data cleaning, molecular representation generation, feature selection, and model construction with class-imbalance-aware training.

**FIGURE 2:**
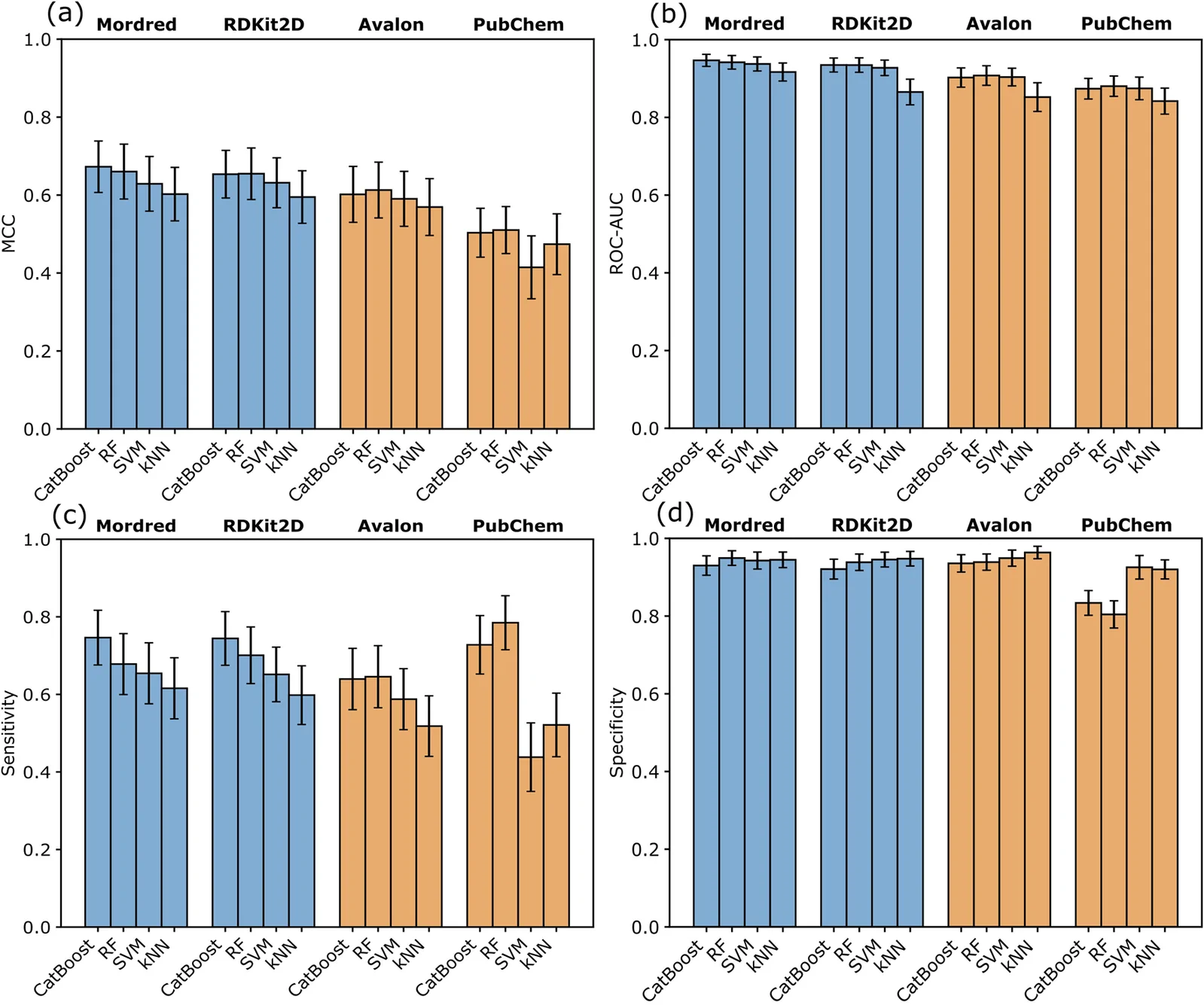
**(A)** MCC, **(B)** ROC-AUC, **(C)** sensitivity, and **(D)** specificity of the models obtained from the 30 iterations of the 5-fold cross-validation on the training set. The four grouped bar charts, shown from left to right, correspond to two continuous molecular descriptor sets (blue; Mordred and RDKit2D descriptors) and two discrete molecular fingerprints (orange; Avalon and PubChem fingerprints). Within each group, the bars (ordered from left to right) represent the performance of the CatBoost, RF, SVM, and kNN models, respectively. Error bars represent the corresponding standard deviations.

The cross-validation performance of all models on the training set is summarized in Figure 2. Complete numerical values for all metrics, including those not shown in the figure, are provided in Supplementary Table S1 in the Supplementary Material. Overall, most models achieved accuracies close to 0.88 and MCC values close to 0.60, indicating moderately strong balanced predictive performance when sensitivity and specificity were considered jointly. For ROC-AUC, most models exceeded 0.90, showing strong discriminatory ability between BSEP inhibitors and non-inhibitors. The sensitivity and specificity results showed a clear imbalance-related pattern. Sensitivity generally ranged from 0.60 to 0.80, whereas specificity was mostly above 0.90. In the 5-fold cross-validation setting, each validation fold contained only approximately 40 positive samples, and sensitivity estimates showed relatively large variability, with standard deviations of approximately 10%–15%. In contrast, specificity was more stable because of the larger number of negative samples. MCC also showed relatively large cross-validation variance, whereas ROC-AUC remained more stable across iterations.

**FIGURE 3:**
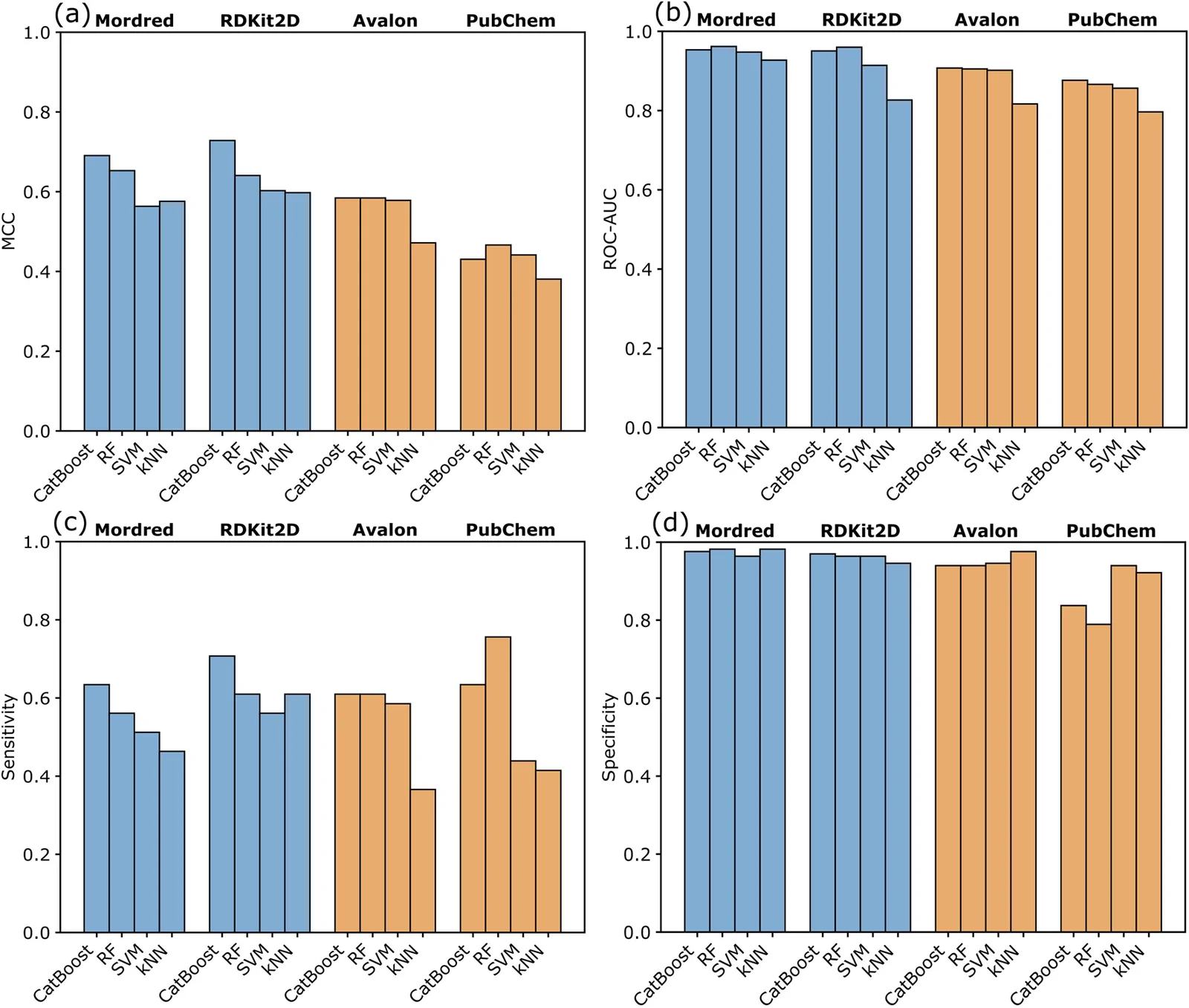
**(A)** MCC, **(B)** ROC-AUC, **(C)** sensitivity, and **(D)** specificity of the models obtained from predictions of the test set. The four grouped bar charts, shown from left to right, correspond to two continuous molecular descriptor sets (blue; Mordred and RDKit2D descriptors) and two discrete molecular fingerprints (orange; Avalon and PubChem fingerprints). Within each group, the bars (ordered from left to right) represent the performance of the CatBoost, RF, SVM, and kNN models, respectively.

**FIGURE 4:**
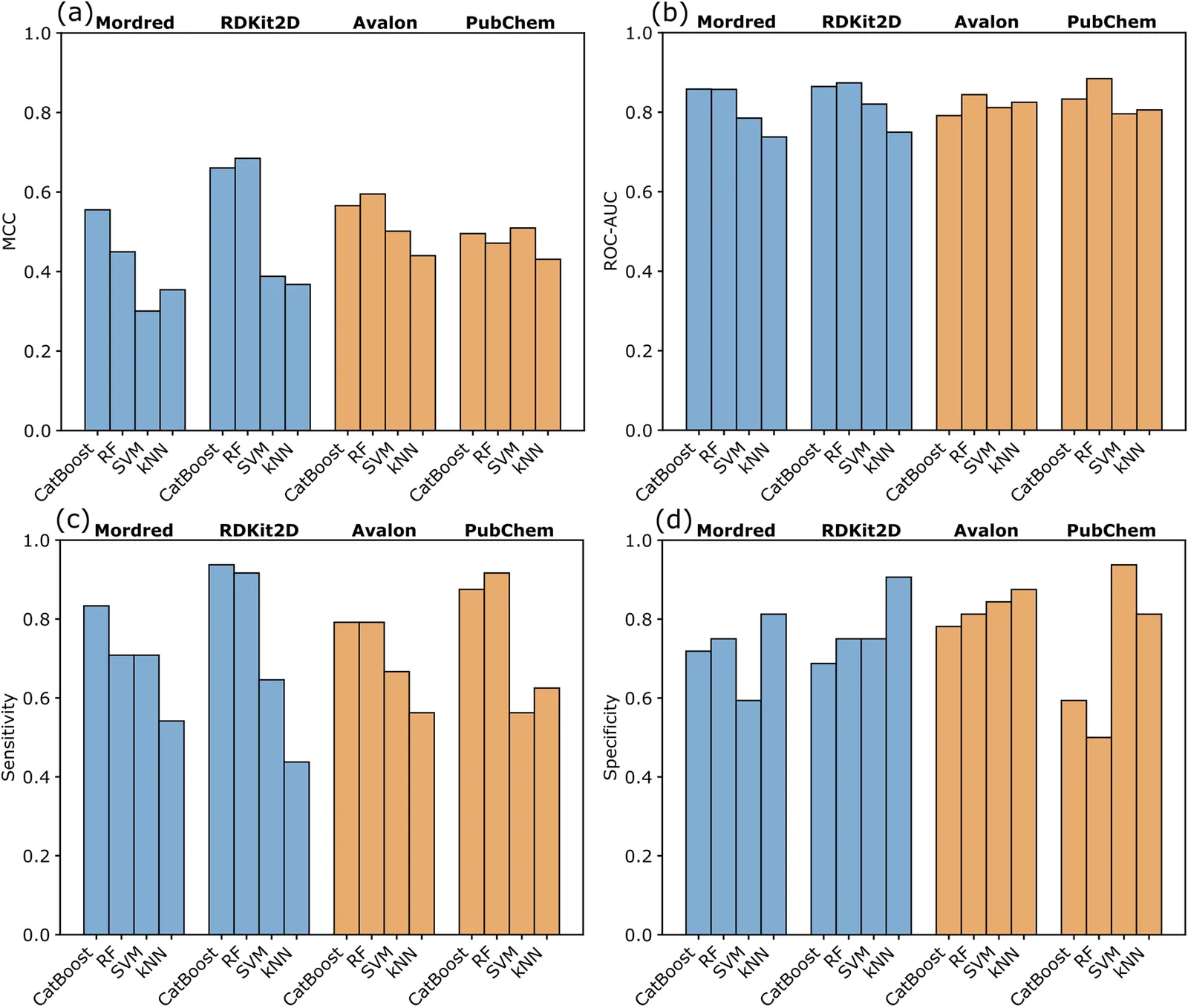
**(A)** MCC, **(B)** ROC-AUC, **(C)** sensitivity, and **(D)** specificity of the models obtained from predictions of the external validation set. The four grouped bar charts, shown from left to right, correspond to two continuous molecular descriptor sets (blue; Mordred and RDKit2D descriptors) and two discrete molecular fingerprints (orange; Avalon and PubChem fingerprints). Within each group, the bars (ordered from left to right) represent the performance of the CatBoost, RF, SVM, and kNN models, respectively.

The performances of all models on the test set and external validation set are shown in Figures 3 and 4, respectively. Complete numerical values for all metrics, including those not shown in the figures, are provided in Supplementary Tables S2 and S3. Test-set performance was generally consistent with the cross-validation results. In contrast, model performance declined on the external validation set which contains 80 compounds, including 48 BSEP inhibitors and 32 non-inhibitors, and the bootstrap confidence intervals were generally wider than those obtained for the test set. Among all tested molecular representations, tree-based models combined with RDKit2D descriptors achieved the strongest external validation performance, with MCC values exceeding 0.65 and ROC-AUC reaching 0.87. Although their confidence intervals overlapped with those of several competing model combinations because of the limited external sample size, paired bootstrap analysis provided additional support for the external advantage of tree-based learning; the results are summarized in Supplementary Table S4. Other descriptor types combined with tree-based algorithms typically produced MCC point estimates around 0.45–0.50, whereas SVM and kNN models performed slightly worse. The external validation set contained more positive than negative samples. Most models showed relatively high sensitivity on this set. For example, tree-based models combined with RDKit2D descriptors or PubChem fingerprints achieved sensitivity values of approximately 0.90, while specificity remained around 0.70–0.80 for most models.

### 3.2 Analysis of Boruta feature selection

**FIGURE 5:**
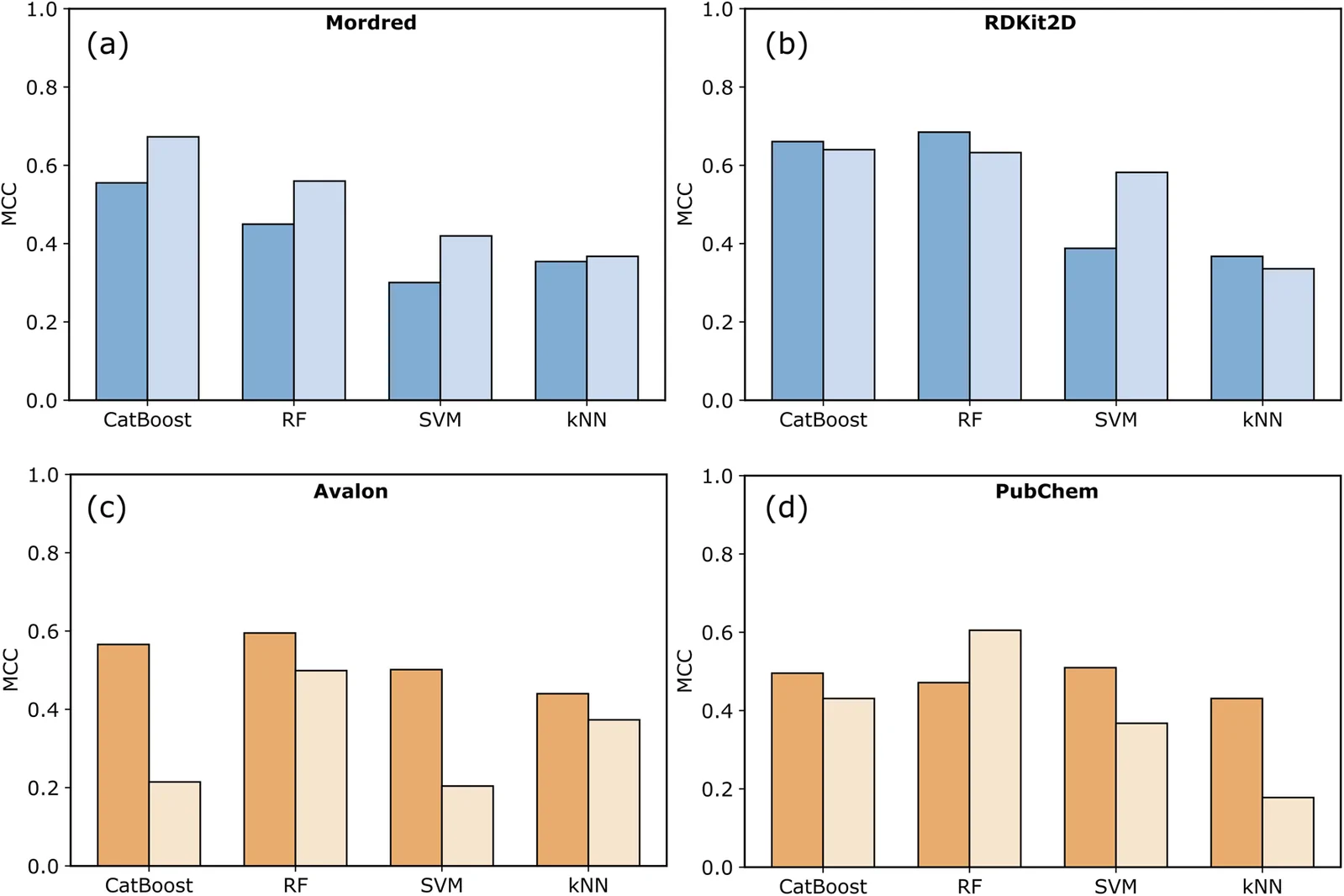
MCC of the models obtained from predictions of the external validation set using two molecular descriptors **(A)** Mordred, **(B)** RDKit2D and two hashed molecular fingerprints **(C)** Avalon, **(D)** PubChem. Performances were grouped with CatBoost, RF, SVM and kNN modeling strategies, respectively. In each group, darker color bar indicates Boruta feature selection was applied, and lighter color bar indicates Boruta was skipped while other feature selection pipeline remained.

To evaluate the practical impact of Boruta feature selection, a controlled comparison was conducted. In the control group, the standard feature preprocessing steps for both continuous molecular descriptors and discrete fingerprints were retained, but Boruta feature selection was omitted. All other modeling procedures were kept identical to those in the experimental group. The numbers of features generated by different molecular representations before and after Boruta selection are summarized in Table 2. The corresponding MCC comparisons between the experimental and control groups on the external validation set are shown in Figure 5, while the MCC comparisons on the training and test sets are provided in Supplementary Figures S1 and S2.

**TABLE 2:** Numbers of features before initial feature selection, before Boruta feature selection and after Boruta feature selection.

Number of features	Before initial feature selection	Before boruta feature selection	After boruta feature selection
Mordred	1613	351	65
RDKit2D	200	138	30
Avalon	512	500	53
PubChem	881	500	41

The feature selection pipeline showed strong dimensionality reduction across different molecular representations, typically retaining only one-tenth or fewer of the original features. For the best-performing RDKit2D descriptors, Boruta reduced the feature number to 30. However, the effect of Boruta selection on external validation performance varied across molecular representations and learning algorithms.

Three major patterns were observed. First, Boruta selection led to a pronounced improvement in generalization performance for certain discrete fingerprints. For example, the Avalon-CatBoost combination improved from limited external predictive utility to practically acceptable performance after Boruta selection. Second, Boruta had a negligible impact on some model settings, such as RDKit2D descriptors combined with tree-based models. Third, Boruta had an unfavorable effect in several cases, mainly for models built with SVM, especially when continuous descriptors were used. In addition to model performance, use of the Boruta-reduced feature sets resulted in a more than five-fold speedup in subsequent model training and hyperparameter optimization.

### 3.3 Applicability domain analysis

Applicability domain (AD) analysis was performed to assess whether the training, test, and external validation compounds were located within the chemical space defined by the training set. Based on the AD definitions described in the Methods section, the numbers of molecules classified as AD-in and AD-out for each dataset were summarized in Table 3.

**TABLE 3:** Number of molecules inside and outside the AD using different molecular representations, of the training set, test set and external validation set respectively.

Descriptors/Fingerprints	Training set		Test set		External validation set	
	AD-in	AD-out	AD-in	AD-out	AD-in	AD-out
Mordred	827	0	196	11	79	1
RDKit2D	827	0	201	6	80	0
Avalon	785	42	199	8	78	2
PubChem	795	32	196	11	79	1

**FIGURE 6:**
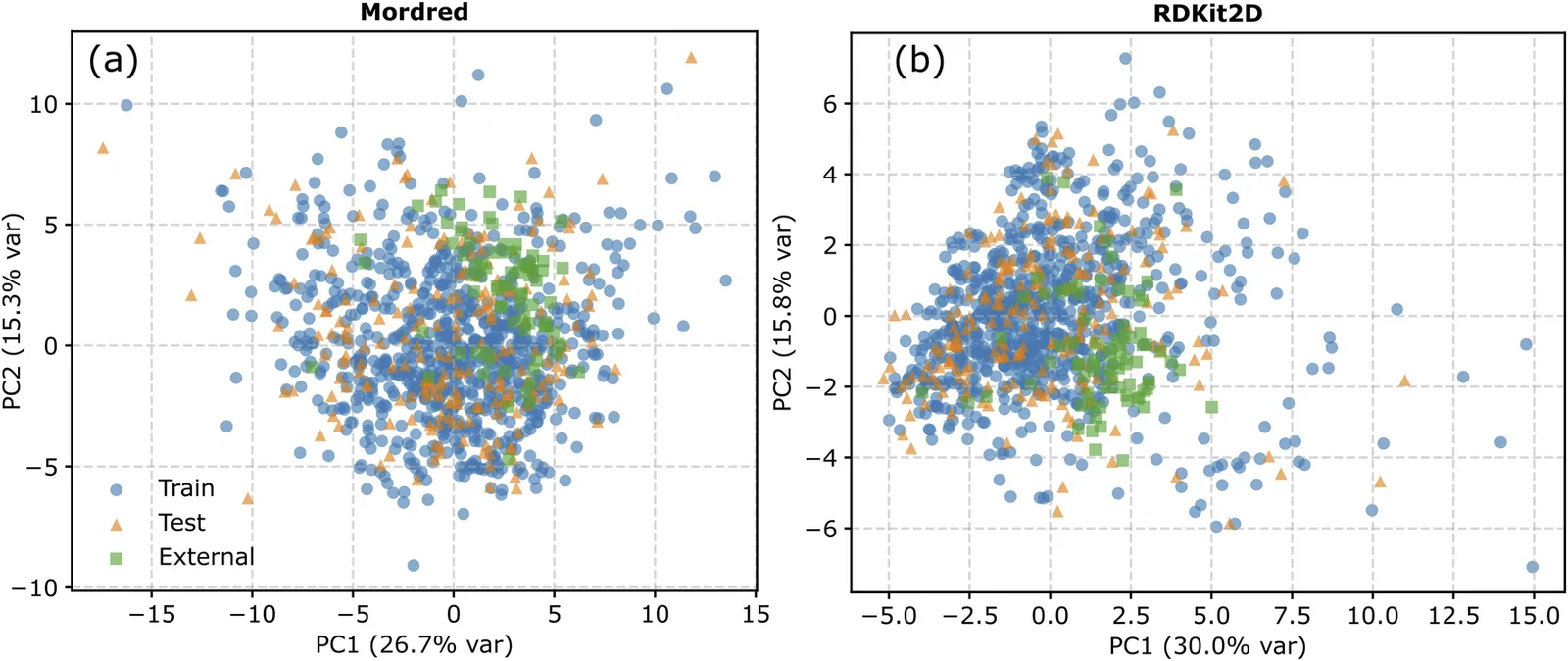
PCA score plot (PC1 vs. PC2) of two molecular descriptors **(A)** Mordred and **(B)** RDKit2D for the training set (blue circles), test set (orange triangles), and external validation set (green square). PCA was fitted on the training set only, and the test/external compounds were projected onto the same principal component space to enable a direct comparison of distributional shifts. Axis labels report the variance explained by each component.

For the two continuous descriptor sets, PCA score plots in the space spanned by the first two principal components are shown in Figure 6. For the two discrete fingerprints, similarity distributions were analyzed using kernel density estimation (KDE) plots, as shown in Supplementary Figure S3. For the test and external validation datasets, these distributions represent the maximum Tanimoto similarity of each compound to the training set. As summarized in Table 3, nearly all compounds in both the test set and the external validation set were classified as AD-in across the examined molecular representations.

The fingerprint-similarity KDE plots for the two discrete fingerprints did not show a pronounced structural covariate shift between the external validation set and the training set. This observation is consistent with the relatively limited performance degradation observed for the two fingerprint-based representations on the external validation set. For the two continuous descriptor sets, most external compounds were also classified as AD-in under the boundary-defined criteria. However, the PCA score plots revealed a clear distributional shift, with the external validation compounds displaced relative to the training compounds in the principal component space. This indicates that, although most external compounds were located within the formal AD boundaries, the internally synthesized compounds in the external validation set showed a different descriptor-derived feature distribution from the training data. This observation is consistent with the more pronounced performance degradation observed for continuous descriptors compared with discrete fingerprints.

To complement the boundary-based analysis, an additional density-based AD assessment based on k-nearest-neighbor distances was performed in the Mordred and RDKit2D descriptor spaces, with the method and complete results provided, including Supplementary Table S8 and related context.

To provide a direct physicochemical overview of the chemical space covered by the three datasets, six commonly used molecular properties were summarized as median and interquartile range (Supplementary Table S5). The training and test sets showed highly similar distributions of molecular weight, cLogP, topological polar surface area, hydrogen-bond donors, hydrogen-bond acceptors, and rotatable bonds, consistent with their stratified random split from the same literature-derived dataset. In contrast, the external validation set was shifted toward compounds with higher molecular weight, cLogP, topological polar surface area, hydrogen-bond acceptor counts, and rotatable-bond counts. These results complement the PCA and fingerprint-based AD analyses by confirming a clear physicochemical shift of the external validation set within the evaluated chemical space, although most compounds remained within the formal AD boundaries. Scaffold diversity and overlap with the training set are summarized in Supplementary Table S6 . The test set retained partial scaffold overlap with the training data, whereas the external validation set was predominantly composed of compounds with training-unseen scaffolds.

### 3.4 Feature importance analysis

**FIGURE 7:**
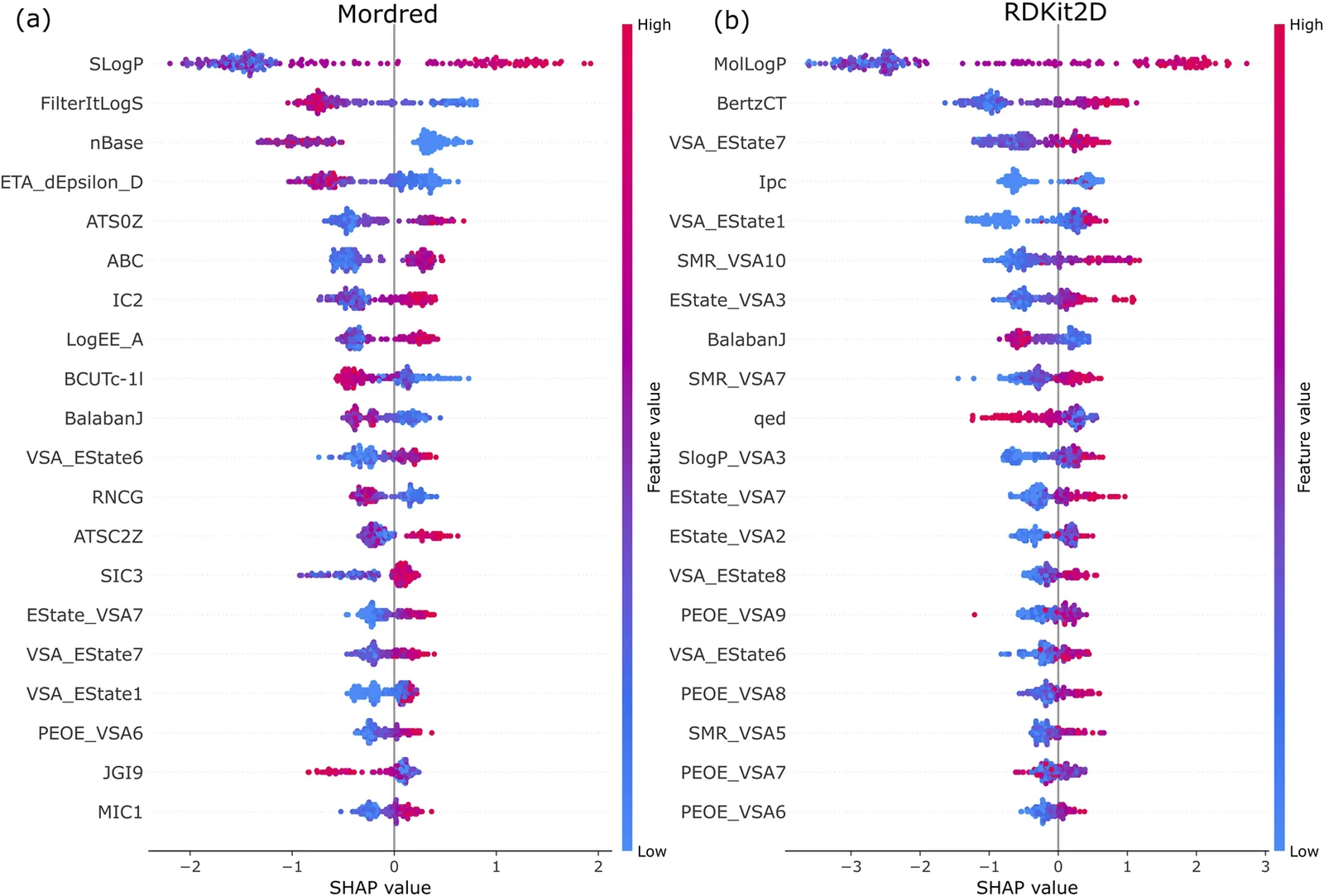
SHAP summary plot for the test set using the **(A)** Mordred and **(B)** RDKit2D descriptor with CatBoost model. Points denote compounds and colors indicate feature values. Positive/negative SHAP values increase/decrease the predicted likelihood of BSEP inhibition. Top 20 features are ordered by mean SHAP values from top to bottom.

SHAP-based interpretability analyses were performed for models built with the continuous Mordred and RDKit2D descriptors on both the test set and the external validation set. The SHAP summary plots for the test set are shown in Figure 7, and the corresponding results for the external validation set are provided in Supplementary Figure S4. The top-ranked features were highly consistent between the two datasets.

Despite differences in descriptor definitions, the leading features from the Mordred and RDKit2D descriptor sets represented similar types of physicochemical information, mainly hydrophobicity/solubility, polarity-related properties, and topological complexity. Among the highly ranked Mordred descriptors, SlogP represents Wildman-Crippen LogP, FilterItLogS reflects solubility, and ETA_dEpsilon_D is related to electronic and polarity-related properties. Several topology-related descriptors, including ATS0Z and the atom-bond connectivity (ABC) index, were also highly ranked. For RDKit2D descriptors, MolLogP was among the important features, while VSA_ESTATE7 and BertzCT also ranked highly, reflecting surface-area/electrotopological and molecular complexity-related information. In addition, nBase showed a pattern in which lower values were associated with a higher predicted probability of BSEP inhibition. Several features highlighted by the SHAP analysis were also consistently retained across all 30 Boruta repetitions. These included SLogP, FilterItLogS, ATS0Z, and ABC among the Mordred descriptors, as well as MolLogP, VSA_EState7, and BertzCT among the RDKit2D descriptors. The complete sets of features selected in every Boruta repetition for each molecular representation are provided in Supplementary Table S7.

For discrete fingerprints, interpretability depended on whether individual bits had explicit structural definitions. Avalon is a hashed fingerprint, and the meaning of a given bit is not clearly defined. Therefore, detailed fragment-level interpretation was not performed for Avalon. In contrast, PubChem fingerprints provide explicit bit definitions, allowing more readable SHAP-based interpretation. The corresponding SHAP summary plot is shown in Supplementary Figure S5. Several highly ranked PubChem bits mainly reflected molecular size and structural complexity. For example, FP_2 indicates the presence of more than 16 hydrogen atoms, FP_12 indicates the presence of more than 16 carbon atoms, and FP_186 denotes the presence of at least two six-membered rings composed only of carbon atoms, either saturated or aromatic. PubChem fingerprints also encoded more specific fragment patterns. For example, FP_643 corresponds to an H-C-C-N-H motif and tended to decrease the predicted inhibition score in the model.

## 4 Discussion

The performance pattern observed in this study reflects the practical challenges of BSEP inhibition modeling under limited and imbalanced data conditions. Because the test set was generated by random splitting from the same data source as the training set, structurally related compounds or similar chemotypes may remain represented in both subsets, resulting in a relatively favorable estimate of predictive performance. In contrast, the external validation set consisted of independently synthesized internal compounds and therefore provided a more stringent assessment of model transferability across data sources and chemical distributions. The observed decrease in external performance should thus be interpreted as a realistic generalization gap rather than solely as model failure. However, because the external validation set was relatively small and imbalanced, individual performance estimates, particularly those derived from the confusion matrix, remain sensitive to the classification of a limited number of compounds.

An additional consideration is that feature selection was performed once using the complete training set before the repeated cross-validation procedure. Therefore, the cross validation results in Figure 2 and Supplementary Table S1 should be interpreted as internal resampling diagnostics of model stability and comparative behavior within a fixed selected feature space, rather than as fully unbiased estimates of out-of-sample generalization. Generalization performance was assessed primarily using the held-out test set and the independent external validation set, neither of which contributed to feature selection or hyperparameter optimization.

The physicochemical-property comparison further supports this interpretation. The training and test sets covered highly similar chemical space with respect to the six examined properties, whereas the external validation compounds were generally larger and more lipophilic, had higher polar surface areas and hydrogen-bond acceptor counts, and were more conformationally flexible. Therefore, the external evaluation involved a measurable chemical-space shift rather than a simple repetition of the physicochemical distribution represented in the training data. The retained predictive performance under this shift supports transferability to an independent project-derived compound collection, although it should not be interpreted as unrestricted generalizability to all chemical classes. The scaffold-overlap analysis was consistent with these chemical-space observations. The same-source test set retained greater scaffold overlap with the training data, whereas the external validation set was predominantly composed of compounds with training-unseen scaffolds, further supporting its role as the more stringent assessment of model transferability across compound collections. Model performance depended on the combination of learning algorithm and molecular representation rather than following a simple relationship with model complexity. Tree-based ensemble models generally achieved stronger external validation performance than SVM and kNN in the present dataset. One possible explanation is that BSEP inhibition is influenced by multiple structural and physicochemical factors, including lipophilicity, molecular size, ionization-related properties, topology, and specific substructural patterns. Interactions among these factors may be nonlinear or threshold-dependent, and tree-based models can capture such relationships without imposing a predefined functional form. However, this result should not be interpreted as evidence that tree-based algorithms are universally superior, because model performance also depends on feature characteristics, sample size, hyperparameter optimization, and chemical-space coverage. Regarding molecular representation, continuous descriptors showed a larger performance decrease on the external validation set than discrete fingerprints. Fingerprints encode local structural patterns and may therefore preserve structural-neighborhood information across datasets, whereas continuous descriptors summarize global physicochemical and topological properties that may be more sensitive to distributional differences between compound collections. Nevertheless, both descriptor and fingerprint based models retained measurable discriminatory ability when combined with tree-based algorithms, with MCC values around 0.50 and ROC-AUC values above 0.85 in most cases. The representation-dependent differences in external performance are further examined through the applicability domain analysis.

The external validation results can be compared with representative BSEP models reported previously. McLoughlin and co-workers (McLoughlin et al., 2021) developed a random forest model using Mordred descriptors and reported an MCC of 0.49 and a ROC-AUC of 0.86, whereas Rodríguez-Pérez et al. (Rodríguez-Pérez and Gerebtzoff, 2021) combined RDKit2D descriptors with XGBoost and reported a validation MCC of 0.51. However, direct comparison across studies should be made cautiously because performance is also affected by differences in assay sources, activity thresholds, data curation, class distribution, and validation-set construction. In the present study, class imbalance was retained and the external validation set consisted of independently synthesized compounds. Therefore, the observed results mainly demonstrate that the proposed workflow maintained useful discriminatory ability when applied to compounds from a distinct project setting, rather than indicating superiority over previously published models.

Although under-sampling and oversampling are commonly used strategies for handling class imbalance, they were not adopted in this study. Under-sampling would further reduce the amount of information available for model training, which is undesirable for a small dataset. Oversampling, especially when synthetic samples are introduced, may increase the risk of overfitting, particularly for discrete molecular fingerprints. Therefore, we focused on molecular feature engineering and class-weight-aware training to mitigate imbalance effects while preserving all original samples. For BSEP-related toxicity prediction, failing to identify potentially harmful compounds may carry greater safety implications than incorrectly flagging some non-inhibitors. Maintaining high sensitivity is therefore particularly important in early-stage safety screening. The best-performing models in this study achieved sensitivity values greater than 0.90 on the external validation set, supporting their potential utility as early screening tools for identifying compounds with possible BSEP inhibition risk.

The effect of Boruta feature selection depended on both the molecular representation and the learning algorithm. Previous QSAR studies have shown that predictive information may be concentrated in a relatively small subset of molecular features rather than distributed across hundreds or thousands of descriptors (Dan et al., 2025; El-Idrissi et al., 2025). This is consistent with the substantial dimensionality reduction observed in the present study, particularly for RDKit2D, for which 30 descriptors remained after Boruta selection. For high-dimensional sparse fingerprints, many bits may be redundant, infrequent, or weakly associated with the endpoint; removing such features can reduce noise and improve the stability of tree-based models, as observed for the Avalon-CatBoost combination. In contrast, RDKit2D descriptors had already undergone preprocessing and contained a smaller set of relatively information-dense variables, leaving less scope for further improvement through Boruta. The performance decrease observed for several SVM models may reflect a mismatch between the selector and the downstream algorithm. Because Boruta identifies features using tree-based importance, the selected subset may not preserve the distance or kernel-space relationships required by SVM. Thus, Boruta should not be regarded as a universally beneficial step, but as a representation- and algorithm-dependent component of the modeling workflow.

Overall, Boruta feature selection appears generally suitable when molecular representations are combined with tree-based models, although its benefit is not universal. Even in cases such as Mordred descriptors paired with random forest, where a small decrease in MCC was observed, external validation performance remained at an acceptable level of approximately 0.45. The computational efficiency gain obtained through Boruta-based dimensionality reduction is also practically important, especially when more complex modeling strategies, such as extensive hyperparameter optimization, are used. From an industrial perspective, QSAR models are often updated iteratively as new compounds are incorporated into the training set during a project. In this context, improved modeling efficiency is advantageous for periodic model retraining.

The AD results suggest that model reliability and chemical-space coverage should be interpreted in a representation-dependent manner. In fingerprint space, the external compounds largely remained within the structural neighborhood covered by the training data, suggesting that predictions based on the two discrete fingerprints were more likely dominated by interpolation rather than strong extrapolation. This may partly explain why fingerprint-based models showed relatively stable performance on the external validation set. In contrast, the continuous descriptor-based PCA analysis revealed a clearer covariate shift for the external validation set. Together with the physicochemical-property distributions summarized in Supplementary Table S5, this result indicates that a range-based AD definition can provide broad coverage but may be insensitive to distributional shifts occurring within the formal boundaries. Newly synthesized compounds generated in an internal laboratory cannot be assumed to perfectly match the distribution of public training compounds, and some degree of performance degradation on the external validation set is therefore expected. However, the models retained acceptable predictive performance on the external dataset, suggesting that moderate distributional shift or limited extrapolation beyond the densely sampled regions of the training descriptor space does not necessarily preclude meaningful predictions. These findings further support that AD assessment is intrinsically dependent on the selected molecular representation. Although most external compounds were classified as AD-in, the extent of covariate shift differed across molecular representations, with a more pronounced displacement observed in continuous descriptor space than in fingerprint space. Therefore, AD results should be interpreted together with model performance and molecular representation characteristics, rather than being used as a standalone criterion for judging prediction reliability.

The SHAP results indicate that lipophilicity, solubility, polarity-related properties, and molecular complexity jointly contribute to BSEP inhibition predictions. The principal descriptors supporting these interpretations were consistently retained across all 30 Boruta repetitions, providing additional support for the robustness of the major physicochemical patterns identified by SHAP. The importance of SlogP and MolLogP is chemically plausible because lipophilic compounds may partition into the canalicular membrane and form favorable hydrophobic contacts within transporter-associated binding regions, thereby increasing their local availability near BSEP. However, lipophilicity should not be considered in isolation: excessive hydrophobicity may reduce aqueous solubility, while an appropriate balance of hydrophobic and polar functionalities may be required for both membrane access and productive interaction with the transporter. Accordingly, FilterItLogS and ETA_dEpsilon_D may reflect complementary aspects of this physicochemical balance. The importance of ATS0Z, the ABC index, BertzCT, and size or ring-related PubChem bits further suggests that molecular size, branching, ring content, and overall topology influence the structural environment recognized by the models. The association between lower nBase values and higher predicted inhibition may reflect a larger neutral fraction or reduced permanent cationic character, which could favor membrane partitioning; nevertheless, the number of basic sites is only an indirect measure of ionization, and this observation should not be interpreted independently of pKa, lipophilicity, and molecular context.

However, SHAP-based interpretation requires caution. SHAP values quantify how individual features influence model output within the fitted model, but they do not establish causal or mechanistic relationships with BSEP inhibition. This limitation is particularly relevant because many physicochemical descriptors are correlated; for example, lipophilicity, solubility, molecular size, and aromaticity may encode overlapping chemical information, causing attribution to be distributed among several features. Similarly, an interpretable PubChem bit may occur preferentially within a broader chemotype, so its SHAP effect may reflect the surrounding structural context rather than the isolated fragment itself. Thus, the negative contribution associated with FP_643 should be regarded as a model-derived hypothesis that may guide subsequent chemical examination, rather than evidence that this motif intrinsically reduces BSEP inhibition. These limitations are especially important in small datasets, where feature rankings may vary with sample composition and correlated structural patterns.

In a broader safety-assessment context, the predicted BSEP-inhibition class or model score could serve as a mechanistic input to a multiparametric evaluation of DILI potential, together with projected human exposure and other relevant liabilities, such as mitochondrial toxicity, reactive-metabolite formation, and inhibition of other hepatobiliary transporters. Compounds predicted to inhibit BSEP could be prioritized for experimental confirmation, after which the measured inhibition potency and estimated unbound exposure could be used to assess the exposure margin. Thus, the present model is intended to support an integrated DILI risk assessment.

In conclusion, this study developed and evaluated a QSAR workflow for predicting BSEP inhibition under practical conditions characterized by limited sample size, high-dimensional molecular representations, and pronounced class imbalance. Models based on continuous descriptors and discrete fingerprints were evaluated using cross-validation, a held-out same source test set, and an independent external validation set comprising compounds synthesized in an industrial R&D setting. Tree-based ensemble models generally showed the strongest external performance, although the results also demonstrated that predictive performance depended on the compatibility between molecular representation, feature-selection strategy, and learning algorithm. Boruta substantially reduced feature dimensionality and improved computational efficiency, but its effect on prediction performance was representation and algorithm-dependent rather than universally beneficial. Applicability-domain and SHAP analyses further provided complementary information on chemical space coverage and the molecular properties influencing model predictions. Collectively, these findings highlight the importance of external validation, representation-aware model development, and cautious interpretation of model applicability. The proposed workflow provides a practical approach for early-stage BSEP risk screening and may inform the development of other toxicity models based on limited and imbalanced datasets.

## Data Availability

The original contributions presented in the study are included in the article/Supplementary Material, further inquiries can be directed to the corresponding author.
