# Supplementary material for: Machine learning prediction of bile salt export pump inhibition with Boruta feature selection in imbalanced data
Source: Front Pharmacol. 2026 Sep 9;17:1911283. doi: 10.3389/fphar.2026.1911283 (PMC13598833; doi:10.3389/fphar.2026.1911283)
Supplement: Supplementary file 1 [file DataSheet1.pdf]

Supplementary Material

BSEP Inhibition Assay for the External Validation Dataset

The inhibitory effects of compounds in the external validation dataset on human BSEP activity were evaluated using membrane vesicles expressing recombinant human BSEP (GenoMembrane). Each reaction contained 50μg of vesicle protein. Test compounds were evaluated at eight concentrations (0.512, 1.28, 3.2, 8, 20, 50, 100, and 200 μM), with two replicate samples at each concentration. Sodium taurocholate (10 μM) was used as the probe substrate. Blank-buffer-treated vesicles served as the negative control, and rifampicin (100 μM) was included as a positive BSEP inhibitor control.

The membrane vesicles were preincubated with the test compounds or controls for 5 min at 37°C. Transport reactions were then initiated by adding sodium taurocholate under parallel ATP and AMP-containing conditions and were continued for 5 min at 37°C. The reactions were terminated using ice-cold Buffer B1 and transferred to 96-well filter plates. After vacuum filtration, the filters were washed five times with 0.2 mL of ice-cold Buffer B1. Vesicle-associated taurocholate was extracted twice from the filters using 50 μL of 80% methanol, and the two extracts were combined. The combined extract was mixed with ice-cold methanol containing an internal standard, followed by centrifugation. The resulting supernatant was analyzed by LC-MS/MS to quantify transported taurocholate.

ATP-dependent BSEP transport was determined by correcting the signal obtained under ATP-containing conditions for the corresponding signal obtained under AMP-containing conditions. Transport activity at each test-compound concentration was expressed relative to the negative control. Concentration-response data were analyzed using GraphPad Prism to determine the percentage inhibition and IC50 value for each compound.

Table S1. Performance metrics of the cross-validation on the training set. Model names are formatted as "Molecular representation_Model".

Model	Accuracy	Sensitivity	Specificity	Balanced Accuracy	MCC	ROC-AUC
Mordred_KNN	0.879 ± 0.020	0.616 ± 0.079	0.945 ± 0.020	0.780 ± 0.038	0.602 ± 0.069	0.917 ± 0.023
Mordred_SVM	0.885 ± 0.021	0.654 ± 0.079	0.943 ± 0.022	0.799 ± 0.039	0.629 ± 0.070	0.937 ± 0.018
Mordred_RF	0.895 ± 0.021	0.678 ± 0.079	0.949 ± 0.019	0.814 ± 0.040	0.660 ± 0.070	0.942 ± 0.017
Mordred_CatBoost	0.893 ± 0.022	0.746 ± 0.071	0.930 ± 0.025	0.838 ± 0.035	0.673 ± 0.066	0.947 ± 0.016
RDKit2D_KNN	0.878 ± 0.019	0.598 ± 0.076	0.948 ± 0.019	0.773 ± 0.037	0.595 ± 0.067	0.865 ± 0.033
RDKit2D_SVM	0.886 ± 0.019	0.651 ± 0.070	0.945 ± 0.019	0.798 ± 0.035	0.632 ± 0.064	0.928 ± 0.020
RDKit2D_RF	0.891 ± 0.021	0.701 ± 0.073	0.938 ± 0.021	0.820 ± 0.036	0.655 ± 0.066	0.935 ± 0.019
RDKit2D_CatBoost	0.885 ± 0.022	0.744 ± 0.069	0.921 ± 0.026	0.833 ± 0.034	0.654 ± 0.061	0.935 ± 0.018
Avalon_KNN	0.874 ± 0.019	0.518 ± 0.078	0.964 ± 0.016	0.741 ± 0.039	0.569 ± 0.073	0.852 ± 0.037
Avalon_SVM	0.877 ± 0.020	0.588 ± 0.079	0.949 ± 0.021	0.768 ± 0.038	0.590 ± 0.070	0.904 ± 0.023
Avalon_RF	0.880 ± 0.021	0.646 ± 0.080	0.939 ± 0.021	0.792 ± 0.040	0.613 ± 0.072	0.908 ± 0.025
Avalon_CatBoost	0.876 ± 0.022	0.640 ± 0.079	0.936 ± 0.022	0.788 ± 0.039	0.602 ± 0.072	0.903 ± 0.025
PubChem_KNN	0.840 ± 0.023	0.521 ± 0.082	0.920 ± 0.025	0.721 ± 0.041	0.474 ± 0.078	0.842 ± 0.034
PubChem_SVM	0.828 ± 0.024	0.438 ± 0.088	0.926 ± 0.030	0.682 ± 0.041	0.415 ± 0.081	0.875 ± 0.029
PubChem_RF	0.800 ± 0.028	0.785 ± 0.070	0.804 ± 0.035	0.795 ± 0.035	0.510 ± 0.060	0.880 ± 0.026
PubChem_CatBoost	0.813 ± 0.026	0.728 ± 0.075	0.834 ± 0.032	0.781 ± 0.037	0.503 ± 0.063	0.874 ± 0.027

Table S2. Performance metrics of the models on the test set with bootstrap 95% confidence intervals. Values are presented as point estimates [95% confidence interval]. Model names are formatted as "Molecular representation_Model".

Model	Accuracy	Sensitivity	Specificity	Balanced Accuracy	MCC	ROC-AUC
Mordred_KNN	0.879 [0.845-0.913]	0.463 [0.317-0.610]	0.982 [0.958-1.000]	0.723 [0.646-0.802]	0.576 [0.426-0.709]	0.927 [0.875-0.969]
Mordred_SVM	0.874 [0.836-0.913]	0.512 [0.366-0.659]	0.964 [0.934-0.988]	0.738 [0.659-0.814]	0.563 [0.406-0.707]	0.948 [0.904-0.979]
Mordred_RF	0.899 [0.865-0.932]	0.561 [0.415-0.707]	0.982 [0.958-1.000]	0.771 [0.695-0.845]	0.653 [0.514-0.777]	0.962 [0.931-0.985]
Mordred_CatBoost	0.908 [0.870-0.942]	0.634 [0.488-0.780]	0.976 [0.952-0.994]	0.805 [0.729-0.875]	0.691 [0.556-0.811]	0.953 [0.920-0.979]
RDKit2D_KNN	0.879 [0.836-0.918]	0.610 [0.463-0.756]	0.946 [0.910-0.976]	0.778 [0.702-0.854]	0.598 [0.453-0.735]	0.827 [0.750-0.897]
RDKit2D_SVM	0.884 [0.845-0.918]	0.561 [0.415-0.707]	0.964 [0.934-0.988]	0.762 [0.683-0.839]	0.603 [0.450-0.731]	0.914 [0.861-0.958]
RDKit2D_RF	0.894 [0.855-0.928]	0.610 [0.463-0.756]	0.964 [0.934-0.988]	0.787 [0.711-0.860]	0.641 [0.501-0.763]	0.960 [0.929-0.983]
RDKit2D_CatBoost	0.918 [0.879-0.952]	0.707 [0.561-0.829]	0.970 [0.940-0.994]	0.839 [0.765-0.906]	0.728 [0.603-0.843]	0.950 [0.912-0.979]
Avalon_KNN	0.855 [0.821-0.889]	0.366 [0.220-0.512]	0.976 [0.952-0.994]	0.671 [0.598-0.747]	0.472 [0.310-0.617]	0.817 [0.733-0.894]
Avalon_SVM	0.874 [0.831-0.913]	0.585 [0.439-0.732]	0.946 [0.910-0.976]	0.766 [0.686-0.842]	0.578 [0.426-0.714]	0.902 [0.847-0.944]
Avalon_RF	0.874 [0.831-0.913]	0.610 [0.463-0.756]	0.940 [0.904-0.976]	0.775 [0.695-0.851]	0.584 [0.433-0.726]	0.905 [0.856-0.947]
Avalon_CatBoost	0.874 [0.831-0.913]	0.610 [0.463-0.756]	0.940 [0.904-0.976]	0.775 [0.696-0.848]	0.584 [0.434-0.720]	0.907 [0.853-0.950]
PubChem_KNN	0.821 [0.778-0.865]	0.415 [0.268-0.585]	0.922 [0.880-0.958]	0.668 [0.592-0.751]	0.381 [0.218-0.545]	0.797 [0.715-0.873]
PubChem_SVM	0.841 [0.797-0.879]	0.439 [0.293-0.585]	0.940 [0.898-0.976]	0.689 [0.610-0.769]	0.441 [0.275-0.597]	0.856 [0.792-0.912]
PubChem_RF	0.783 [0.725-0.836]	0.756 [0.610-0.878]	0.789 [0.723-0.849]	0.773 [0.697-0.842]	0.466 [0.337-0.591]	0.866 [0.809-0.915]
PubChem_CatBoost	0.797 [0.739-0.850]	0.634 [0.488-0.780]	0.837 [0.777-0.892]	0.736 [0.657-0.818]	0.431 [0.286-0.579]	0.876 [0.822-0.924]

Table S3. Performance metrics of the models on the external validation set with bootstrap 95% confidence intervals. Values are presented as point estimates [95% confidence interval]. Model names are formatted as "Molecular representation_Model".

**Model**	**Accuracy**	**Sensitivity**	**Specificity**	**Balanced Accuracy**	**MCC**	**ROC-AUC**
Mordred_KNN	0.650 [0.550-0.750]	0.542 [0.396-0.688]	0.813 [0.688-0.938]	0.677 [0.578-0.776]	0.354 [0.163-0.546]	0.738 [0.631-0.839]
Mordred_SVM	0.663 [0.563-0.763]	0.708 [0.583-0.833]	0.594 [0.437-0.750]	0.651 [0.542-0.755]	0.301 [0.087-0.508]	0.785 [0.682-0.876]
Mordred_RF	0.725 [0.625-0.825]	0.708 [0.583-0.833]	0.750 [0.594-0.906]	0.729 [0.625-0.823]	0.450 [0.249-0.643]	0.857 [0.765-0.936]
Mordred_CatBoost	0.788 [0.700-0.875]	0.833 [0.729-0.938]	0.719 [0.563-0.875]	0.776 [0.677-0.870]	0.555 [0.364-0.740]	0.858 [0.771-0.934]
RDKit2D_KNN	0.625 [0.538-0.713]	0.438 [0.292-0.583]	0.906 [0.781-1.000]	0.672 [0.589-0.760]	0.367 [0.197-0.529]	0.750 [0.641-0.852]
RDKit2D_SVM	0.688 [0.588-0.788]	0.646 [0.500-0.771]	0.750 [0.594-0.906]	0.698 [0.599-0.797]	0.388 [0.194-0.585]	0.820 [0.727-0.905]
RDKit2D_RF	0.850 [0.775-0.925]	0.917 [0.833-0.979]	0.750 [0.594-0.906]	0.833 [0.750-0.917]	0.685 [0.523-0.844]	0.874 [0.782-0.954]
RDKit2D_CatBoost	0.838 [0.763-0.913]	0.938 [0.854-1.000]	0.688 [0.531-0.844]	0.813 [0.724-0.896]	0.661 [0.496-0.817]	0.865 [0.772-0.947]
Avalon_KNN	0.688 [0.588-0.775]	0.563 [0.417-0.708]	0.875 [0.750-0.969]	0.719 [0.620-0.807]	0.440 [0.255-0.615]	0.825 [0.719-0.915]
Avalon_SVM	0.738 [0.638-0.838]	0.667 [0.542-0.792]	0.844 [0.719-0.969]	0.755 [0.661-0.849]	0.502 [0.317-0.684]	0.812 [0.710-0.902]
Avalon_RF	0.800 [0.713-0.888]	0.792 [0.667-0.896]	0.813 [0.656-0.938]	0.802 [0.708-0.885]	0.595 [0.411-0.767]	0.844 [0.752-0.924]
Avalon_CatBoost	0.788 [0.700-0.875]	0.792 [0.667-0.896]	0.781 [0.625-0.906]	0.786 [0.693-0.875]	0.566 [0.382-0.743]	0.791 [0.684-0.893]
PubChem_KNN	0.700 [0.600-0.800]	0.625 [0.500-0.771]	0.813 [0.656-0.938]	0.719 [0.620-0.813]	0.431 [0.237-0.617]	0.806 [0.694-0.901]
PubChem_SVM	0.713 [0.625-0.800]	0.563 [0.417-0.708]	0.938 [0.844-1.000]	0.750 [0.667-0.833]	0.510 [0.350-0.655]	0.796 [0.696-0.885]
PubChem_RF	0.750 [0.663-0.838]	0.917 [0.833-0.979]	0.500 [0.313-0.688]	0.708 [0.609-0.802]	0.471 [0.264-0.658]	0.884 [0.807-0.949]
PubChem_CatBoost	0.763 [0.675-0.850]	0.875 [0.771-0.958]	0.594 [0.406-0.750]	0.734 [0.635-0.828]	0.495 [0.295-0.685]	0.833 [0.742-0.913]

Paired Bootstrap Comparison of External MCC Estimates

The model-specific bootstrap confidence intervals for the external validation set were relatively wide, primarily because the dataset contained only 80 compounds, including 48 inhibitors and 32 non-inhibitors. However, overlap between the confidence intervals of individual models does not directly determine whether two models differ, because all models were evaluated on the same compounds and their performance estimates were therefore paired. To provide a more direct assessment of the algorithm-dependent differences, paired stratified bootstrap comparisons were performed between each tree-based model and each non-tree model within the same molecular representation. In each of 5,000 bootstrap iterations, the positive and negative compounds were resampled separately with replacement while preserving the original class counts, and the same sampled compound indices were applied to both models. The MCC difference was calculated as the MCC of the tree-based model minus that of the non-tree model, and its 95% confidence interval was defined by the 2.5th and 97.5th percentiles of the paired difference distribution.

Table S4. Paired bootstrap comparisons of tree-based and non-tree models within each molecular representation on the external validation set.

Molecular representation	Tree-based model	Tree-model MCC	Non-tree model	Non-tree model MCC	ΔMCC (tree − non-tree) [95% CI]
Mordred	RF	0.450	kNN	0.354	0.095 [−0.095, 0.289]
Mordred	RF	0.450	SVM	0.301	0.149 [−0.017, 0.324]
Mordred	CatBoost	0.555	kNN	0.354	0.201 [0.005, 0.406]
Mordred	CatBoost	0.555	SVM	0.301	0.255 [0.100, 0.431]
RDKit2D	RF	0.685	kNN	0.367	0.317 [0.117, 0.517]
RDKit2D	RF	0.685	SVM	0.388	0.297 [0.092, 0.508]
RDKit2D	CatBoost	0.661	kNN	0.367	0.293 [0.077, 0.510]
RDKit2D	CatBoost	0.661	SVM	0.388	0.273 [0.039, 0.503]
Avalon	RF	0.595	kNN	0.440	0.155 [0.012, 0.295]
Avalon	RF	0.595	SVM	0.502	0.093 [−0.047, 0.232]
Avalon	CatBoost	0.566	kNN	0.440	0.126 [−0.035, 0.283]
Avalon	CatBoost	0.566	SVM	0.502	0.064 [−0.060, 0.185]
PubChem	RF	0.471	kNN	0.431	0.041 [−0.160, 0.241]
PubChem	RF	0.471	SVM	0.510	−0.038 [−0.253, 0.169]
PubChem	CatBoost	0.495	kNN	0.431	0.065 [−0.153, 0.281]
PubChem	CatBoost	0.495	SVM	0.510	−0.014 [−0.217, 0.183]

RF and CatBoost were classified as tree-based models, whereas kNN and SVM were classified as non-tree models. Positive ΔMCC values favor the tree-based model.

Among the 16 representation-matched comparisons, 14 produced positive ΔMCC point estimates, with a median improvement of 0.138 in favor of the tree-based models. The clearest and most consistent advantage was observed for RDKit2D descriptors: both RF and CatBoost outperformed both kNN and SVM, with ΔMCC values ranging from 0.273 to 0.317 and all four paired confidence intervals excluding zero. The PubChem results were mixed, with the tree-based models outperforming kNN but not SVM and all corresponding intervals including zero. These paired comparisons therefore support the general tendency of tree-based models to achieve stronger external MCC performance for most molecular representations, while confirming that the advantage was dependent on the molecular representation and was not universal.

Table S5. Physicochemical property distributions of the training, test, and external validation datasets. Values are presented as median [interquartile range]. MW, molecular weight; cLogP, calculated logP; TPSA, topological polar surface area; HBD, number of hydrogen-bond donors; HBA, number of hydrogen-bond acceptors; RotB, number of rotatable bonds.

Dataset	MW(g/mol)	cLogP	TPSA($Å^2$)	HBD	HBA	RotB
Training	325.50 [247.26-414.89]	2.36 [0.96-3.66]	74.60 [49.55-106.39]	2.00 [1.00-3.00]	4.00 [3.00-6.00]	4.00 [2.00-7.00]
Test set	311.41 [244.29-420.52]	2.36 [0.51-3.53]	74.28 [47.38-105.66]	2.00 [1.00-3.00]	4.00 [3.00-6.00]	4.00 [2.00-6.00]
External validation	484.65 [435.27-520.83]	4.23 [3.36-5.09]	94.74 [79.81-113.68]	2.00 [1.00-2.00]	6.00 [5.00-8.00]	6.00 [5.00-8.00]

Table S6. Bemis-Murcko scaffold diversity and compound-level scaffold overlap with the training set in the test and external validation datasets. Training-shared denotes compounds whose Bemis–Murcko scaffold was present in the training set, whereas unseen denotes compounds whose scaffold was absent from the training set. The values in these two columns refer to numbers of compounds rather than numbers of unique scaffolds.

Dataset	No. of compounds	Unique scaffolds	Training-shared	Unseen
Test set	207	166	100	107
External set	80	59	10	70

Table S7. Features consistently selected across all 30 Boruta repetitions for each molecular representation. A feature was considered consistently selected when it was retained in every Boruta repetition.

Mordred	RDKit2D	Avalon	PubChem
ABC	BalabanJ	FP_1852	FP_2
LogEE_A	BertzCT	FP_1238	FP_3
VE1_A	EState_VSA3	FP_120	FP_12
VR1_A	EState_VSA7	FP_23	FP_185
nAromAtom	FpDensityMorgan1	FP_1731	FP_186
ATS0Z	Ipc	FP_806	FP_188
AATS0s	MaxAbsEStateIndex	FP_51	FP_192
AATS0se	MolLogP	FP_49	FP_195
AATS1i	NumAromaticRings	FP_1656	FP_482
ATSC1dv	PEOE_VSA6	FP_1543	FP_490

ATSC1d	PEOE_VSA7	FP_455	FP_516
ATSC1Z	PEOE_VSA8	FP_1402	FP_524
AATSC0c	PEOE_VSA9	FP_1343	FP_539
AATSC1c	RingCount	FP_1760	FP_556
AATSC0v	SMR_VSA10	FP_790	FP_599
AATSC1se	SMR_VSA5	FP_885	FP_640
MATS1se	SMR_VSA7	FP_818	FP_660
GATS1s	SlogP_VSA3	FP_466	FP_678
GATS1se	VSA_EState1	FP_1475	FP_696
BCUTc-1l	VSA_EState6	FP_1191	
BCUTdv-1l	VSA_EState7	FP_328	
BalabanJ	VSA_EState8	FP_1375	
RNCG	qed	FP_1004	
C2SP2		FP_1047	
Xch-6d		FP_1479	
Xch-6dv		FP_290	
Xc-3dv		FP_1244	
Xc-5dv		FP_829	
SpMAD_Dt			
NaaCH			
NaasC			
AETA_alpha			
AETA_eta			
AETA_eta_F			
ETA_dAlpha_B			
ETA_dEpsilon_D			
IC2			
CIC0			
MIC1			
FilterItLogS			
PEOE_VSA6			
PEOE_VSA7			
EState_VSA3			
EState_VSA7			
VSA_EState6			
nRing			
n6Ring			
SLogP			
JGI9			
JGI10			

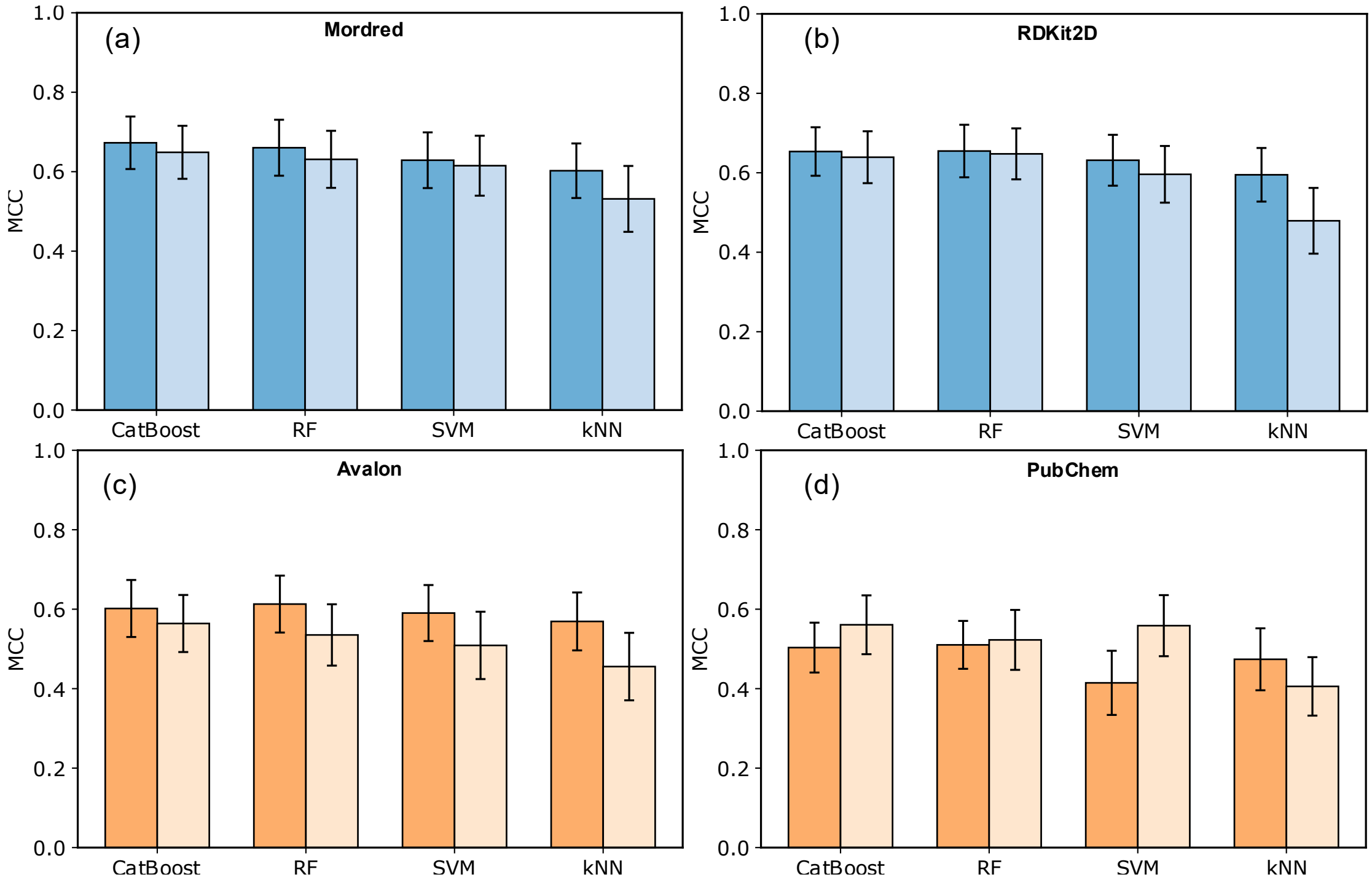

Figure S1. MCC of the models obtained from predictions of the 30 iterations of the 5-fold cross-validation on the training set using two molecular descriptors (a)Mordred, (b)RDKit2D and two hashed molecular fingerprints (c)Avalon, (d)PubChem. Performances were grouped with CatBoost, RF, SVM and kNN modelling strategies, respectively. In each group, darker color bar indicates Boruta feature selection was applied, and lighter color bar indicates Boruta was skipped while other feature selection pipeline remained.

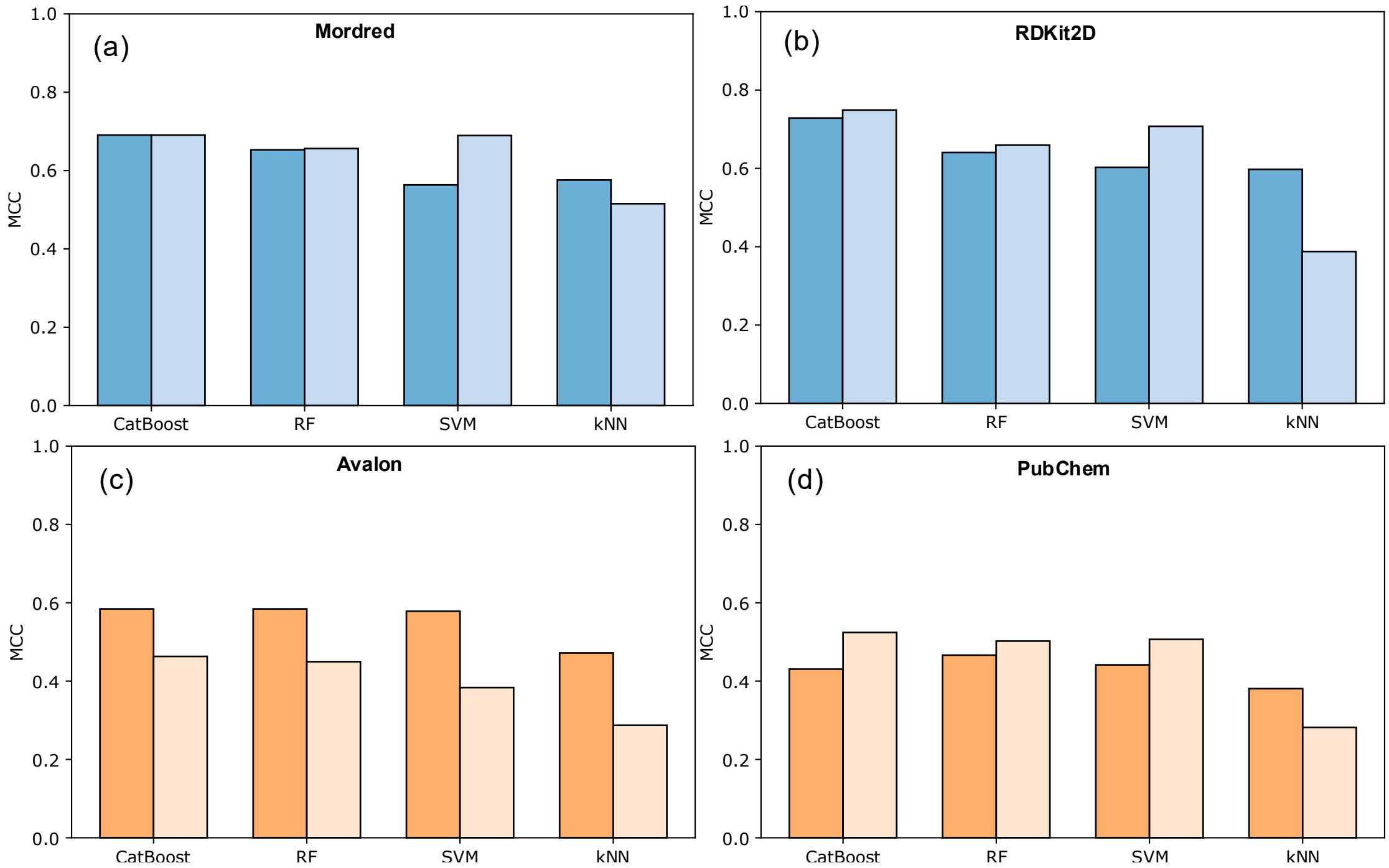

Figure S2. MCC of the models obtained from predictions of the test set using two molecular descriptors (a)Mordred, (b)RDKit2D and two hashed molecular fingerprints (c)Avalon, (d)PubChem. Performances were grouped with CatBoost, RF, SVM and kNN modelling strategies, respectively. In each group, darker color bar indicates Boruta feature selection was applied, and lighter color bar indicates Boruta was skipped while other feature selection pipeline remained.

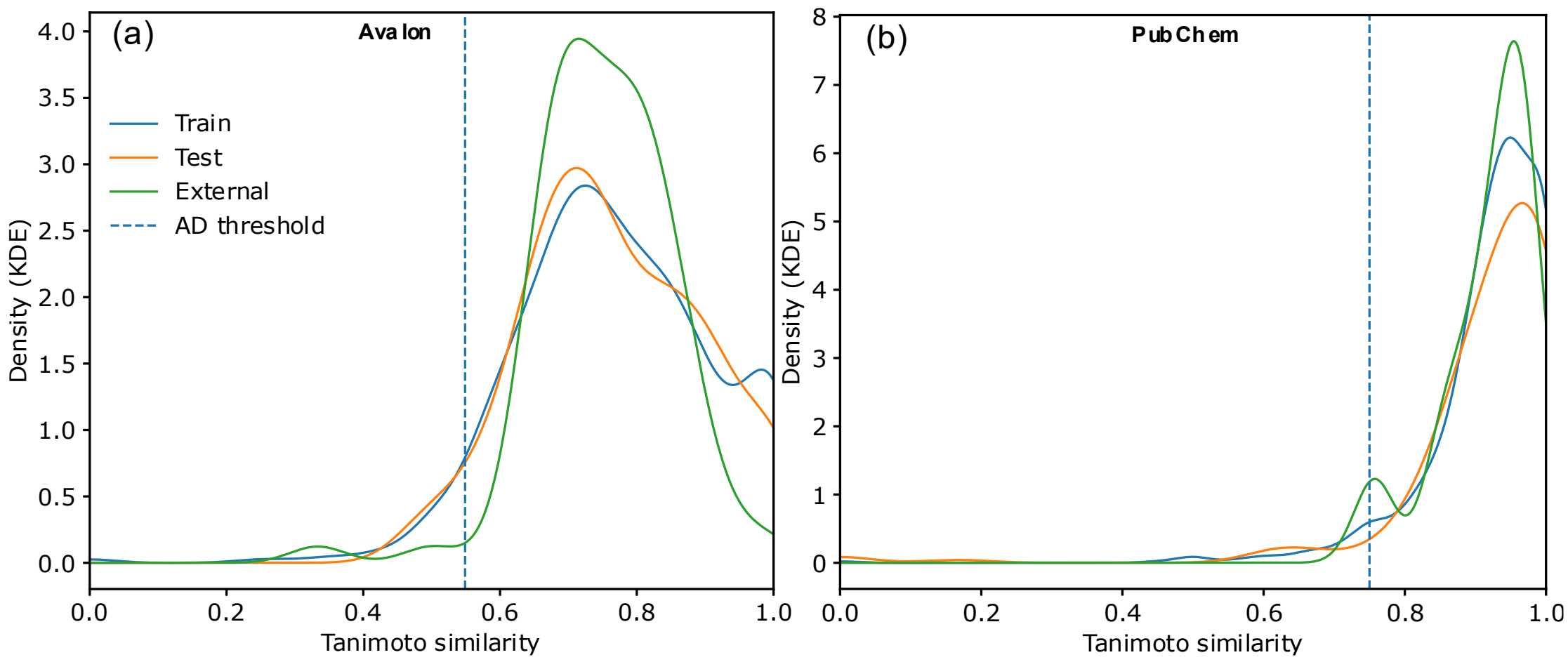

Figure S3. Distributions of nearest-neighbor maximum Tanimoto similarities visualized by KDE, for (a)Avalon and (b)PubChem fingerprints. Blue line represents the distribution of maximum Tanimoto similarity of each compound to its nearest neighbor within the training set. For the test(orange) and external validation(green) sets, the distributions represent the maximum Tanimoto similarity of each compound to the training set. The dashed vertical line denotes the AD threshold derived from the 5th percentile of the training-set nearest-neighbor similarity distribution.

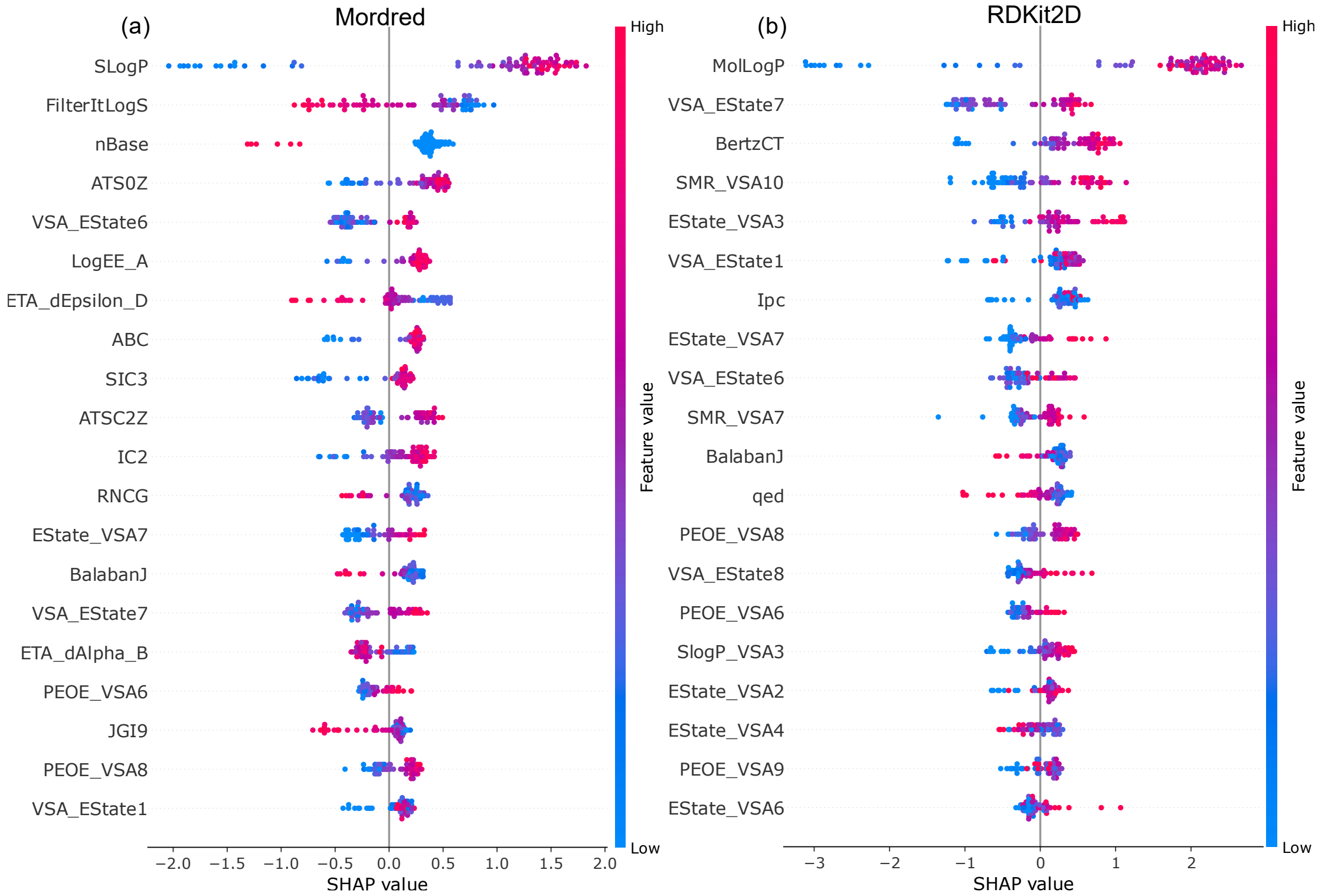

Figure S4. SHAP summary plot for the external validation set using the (a)Mordred and (b)RDKit2D descriptor with CatBoost model. Points denote compounds and colors indicate feature values. Positive/negative SHAP values increase/decrease the predicted likelihood of BSEP inhibition. Top 20 features are ordered by mean SHAP values from top to bottom.

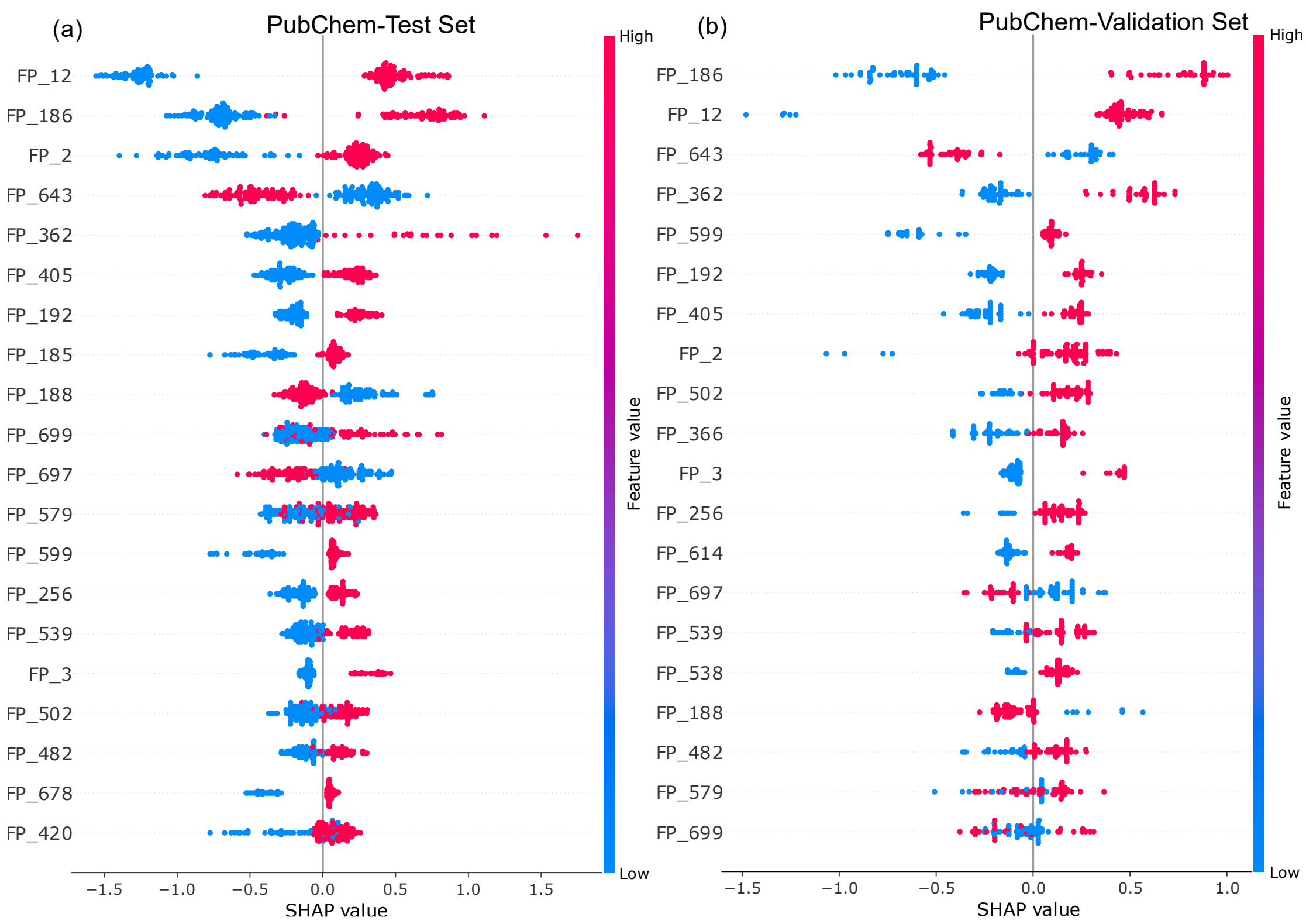

Figure S5. SHAP summary plot for the (a)test set and (b)external validation set using PubChem molecular fingerprints with CatBoost model. Points denote compounds and colors indicate feature values. Positive/negative SHAP values increase/decrease the predicted likelihood of BSEP inhibition. Top 20 features are ordered by mean SHAP values from top to bottom.

Density-Based Applicability Domain Analysis

To complement the range-based applicability-domain analysis, a density-based AD was evaluated in the continuous Mordred and RDKit2D descriptor spaces. For each representation, descriptors were standardized using the mean and standard deviation of the training set, and principal component analysis was fitted using the training data only. Principal components explaining 95% of the cumulative variance were retained, and the test and external validation compounds were projected into the same PCA space. Local density was represented by the mean Euclidean distance from each compound to its 20 nearest training-set neighbors. For training compounds, the compound itself was excluded from the neighbor calculation. The 95th percentile of the training-set mean-distance distribution was used as the AD threshold. Compounds with distances not exceeding this threshold were classified as AD-in, whereas compounds above the threshold were classified as AD-out.

Table S8. Density-based applicability-domain assessment in the Mordred and RDKit2D descriptor spaces. The median distance represents the median of the compound-level mean Euclidean distances to the 20 nearest training set neighbors. The AD threshold was defined as the 95th percentile of the corresponding training set distance distribution.

Representation	Dataset	AD-in, n (%)	AD-out, n (%)	Median distance
Mordred	Test	193 (93.2%)	14 (6.8%)	4.618
Mordred	External	78 (97.5%)	2 (2.5%)	5.159
RDKit2D	Test	198 (95.7%)	9 (4.3%)	2.949
RDKit2D	External	79 (98.8%)	1 (1.2%)	3.766

Most compounds in both evaluation datasets remain within the density-based AD boundary. However, the external validation set showed higher median nearest-neighbor distances than the test set in both descriptor spaces, increasing from 4.618 to 5.159 for Mordred and from 2.949 to 3.766 for RDKit2D. Thus, the continuous density measure detected a greater separation of the external compounds from the training data, although this shift was not sufficient to classify most compounds as AD-out under the 95th-percentile boundary. This pattern was consistent with the range-based AD analysis reported in the main text: both definitions indicated high nominal coverage of the external validation set, while the distance distributions provided additional evidence of the covariate shift identified by the PCA and physicochemical-space analyses.
